# Supplementary material for: Replicate sequencing libraries are important for quantification of allelic imbalance
Source: Nat Commun. 2021 Jun 7;12:3370. doi: 10.1038/s41467-021-23544-8 (PMC8184992; doi:10.1038/s41467-021-23544-8)
Supplement: Supplementary file 1 — Supplementary Information [file 41467_2021_23544_MOESM1_ESM.pdf]

Supplementary Information

for Mendelevich et al., "Replicate sequencing libraries are important for quantification of allelic imbalance"

Contents

. Supplementary Figures . **2**

Supplementary Figure S 1. Different ways of sampling uncover different fractions of technical AI overdispersion. 3

Supplementary Figure S 2. Comparison of agreement between technical replicates in coverage and in AI. 4

Supplementary Figure S 3. Effect of QCC correction on concordance between results of AI imbalance test. 5

Supplementary Figure S 4. Concordance between replicates when using different tools for analyzing allele-specific expression. 6

Supplementary Figure S 5. Principal stages of RNA-seq experiment and data analysis. 7

Supplementary Figure S 6. Assignment of allele-uninformative reads to haplotypes leads to increase in AI overdispersion. 8

Supplementary Figure S 7. QCC captures simulated AI overdispersion. 9

Supplementary Figure S 8. Sources of AI overdispersion: impact of in-silico sampling and repeated sequencing runs (physical library sampling). 11

Supplementary Figure S 9. Sources of AI overdispersion: impact of deduplication. 12

Supplementary Figure S 10. Goodness of fit - R^2 for observed and expected quantiles. 13

Supplementary Figure S 11. Goodness of fit - QQ plots. 14

Supplementary Figure S 12. Goodness of fit - at a single-gene level, AI standard deviation meets expectations. 15

Supplementary Figure S 13. Expectation-maximization fitting of allelic imbalance with Beta-Binomial mixture distribution. 16

Supplementary Figure S 14. Relation between QCC and accounting for AI overdispersion using Beta-Binomial distribution with sample-specific overdispersion parameter ρ. 17

Supplementary Figure S 15. AI overdispersion remains experiment-specific when calculated for individual SNPs. 18

Supplementary Figure S 16. Impact of QCC value on analysis of allele-specific expression in an example GTEx dataset. 19

. Supplementary Tables . **20**

Supplementary Table S 1. RNA-seq datasets analyzed in this study 21

Supplementary Table S 2. Analysis of technical replicates of RNA-seq from human cell lines 22

Supplementary Table S 3. Overdispersion and other features for RNA-seq from mouse neuronal progenitor cells 23

. Supplementary Notes . **24**

Supplementary Note S 1. Is one technical replicate sufficient to separate AI signal from noise? 25

Supplementary Note S 2. Accounting for overdispersion leads to the expected bimodal distribution of AI values in discordant AI calls. 30

Supplementary Note S 3. Genes with different underlying AI have different impact on the overall signal variance. 33

Supplementary Note S 4. We expect nearly zero genes with false positive AI, when we estimate AI and CI from two replicates and then calculate AI from six replicates. 34

Supplementary Note S 5. Statistical power of the QCC-corrected test. 36

Supplementary Note S 6. Worked example of QCC calculation, starting from fastq. 39

Supplementary Note S 7. Worked example of AI differential analysis for two samples. 39

. Supplementary Figures .

for Mendelevich et al., *"Replicate sequencing libraries are important for quantification of allelic imbalance"*

Contents

Supplementary Figure S 1. Different ways of sampling uncover different fractions of technical AI overdispersion. . . . 3
Supplementary Figure S 2. Comparison of agreement between technical replicates in coverage and in AI. . 4
Supplementary Figure S 3. Effect of QCC correction on concordance between results of AI imbalance test. 5
Supplementary Figure S 4. Concordance between replicates when using different tools for analyzing allele-specific expression. . . . 6
Supplementary Figure S 5. Principal stages of RNA-seq experiment and data analysis. . . . 7
Supplementary Figure S 6. Assignment of allele-uninformative reads to haplotypes leads to increase in AI overdispersion. . . . 8
Supplementary Figure S 7. QCC captures simulated AI overdispersion. . . . 9
Supplementary Figure S 8. Sources of AI overdispersion: impact of in-silico sampling and repeated sequencing runs (physical library sampling). . . . 11
Supplementary Figure S 9. Sources of AI overdispersion: impact of deduplication. . . . 12
Supplementary Figure S 10. Goodness of fit - R^2 for observed and expected quantiles. . . . 13
Supplementary Figure S 11. Goodness of fit - QQ plots. . . . 14
Supplementary Figure S 12. Goodness of fit - at a single-gene level, AI standard deviation meets expectations. 15
Supplementary Figure S 13. Expectation-maximization fitting of allelic imbalance with Beta-Binomial mixture distribution. . . . 16
Supplementary Figure S 14. Relation between QCC and accounting for AI overdispersion using Beta-Binomial distribution with sample-specific overdispersion parameter ρ. . . . 17
Supplementary Figure S 15. AI overdispersion remains experiment-specific when calculated for individual SNPs. . . . 18
Supplementary Figure S 16. Impact of QCC value on analysis of allele-specific expression in an example GTEx dataset. . . . 19

Supplementary Figure S 1. Different ways of sampling uncover different fractions of technical AI overdispersion.

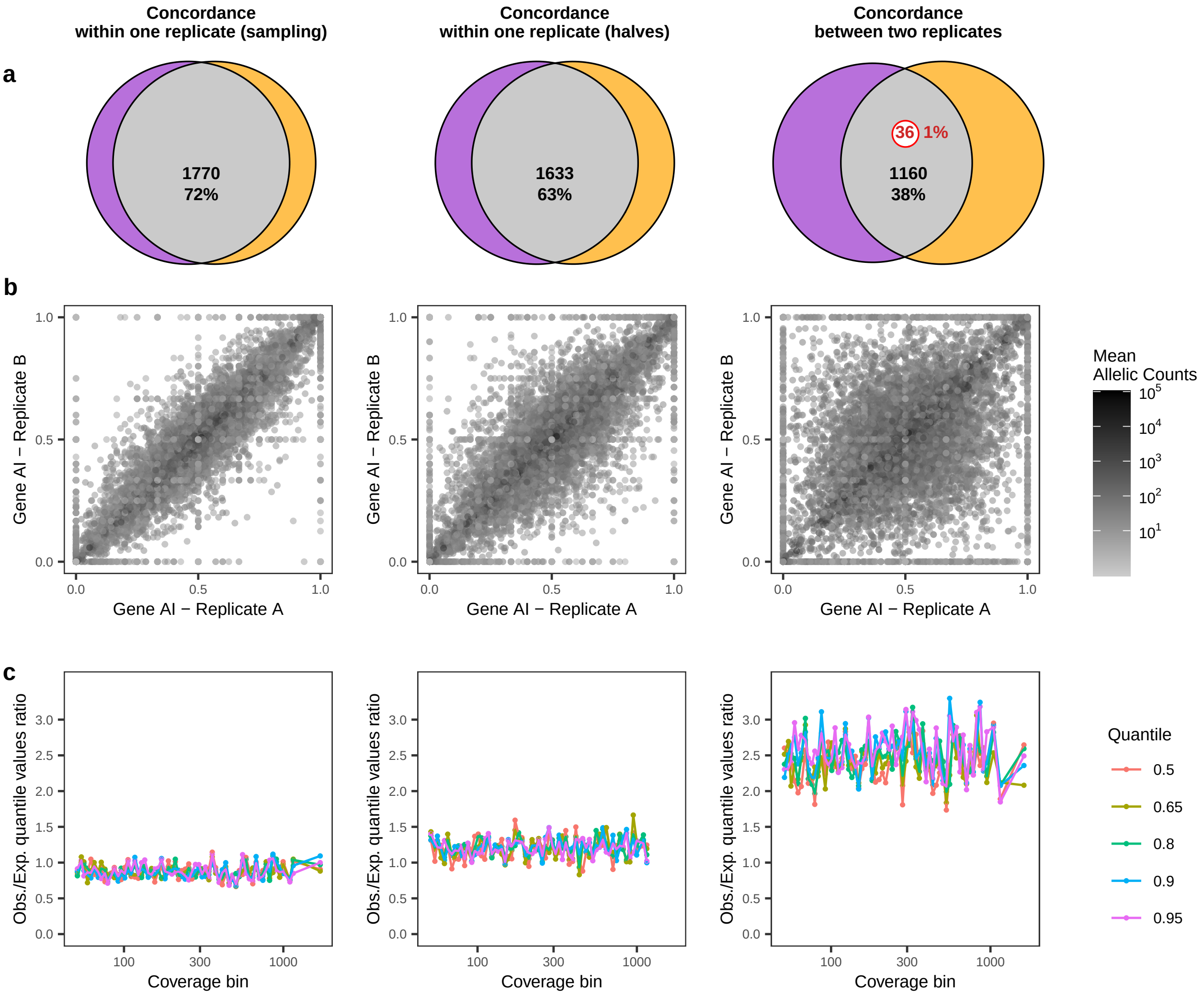

Figure S 1: **Different ways of sampling uncover different fractions of technical AI overdispersion.**

Left to right: Different sampling procedures resulting in sets A and B of 25M reads each sampled from replicates in Experiment 3. **Left:** Sets A and B independently subsampled from 52M reads in the same replicate (A and B are in a binomial relationship with each other); **Middle:** Sets A and B obtained by splitting in half 50M reads from the same RNA-seq library; **Right:** Sets A and B sampled from two technical replicate libraries.
a: Euler diagrams of concordance of "allelically imbalanced" genes (H_0: AI $= 0.5$ is rejected by binomial test; $p = 0.05$ with Bonferroni correction). Same analysis as in **Fig.2c**.
b: Comparison of genes' AI values in the same data as panel **a**.
c: Ratios of observed and expected values of ΔAI for five example quantiles (listed on the right) across coverage bins for the same data (compare to **Fig.3f**)

Supplementary Figure S 2. Comparison of agreement between technical replicates in coverage and in AI.

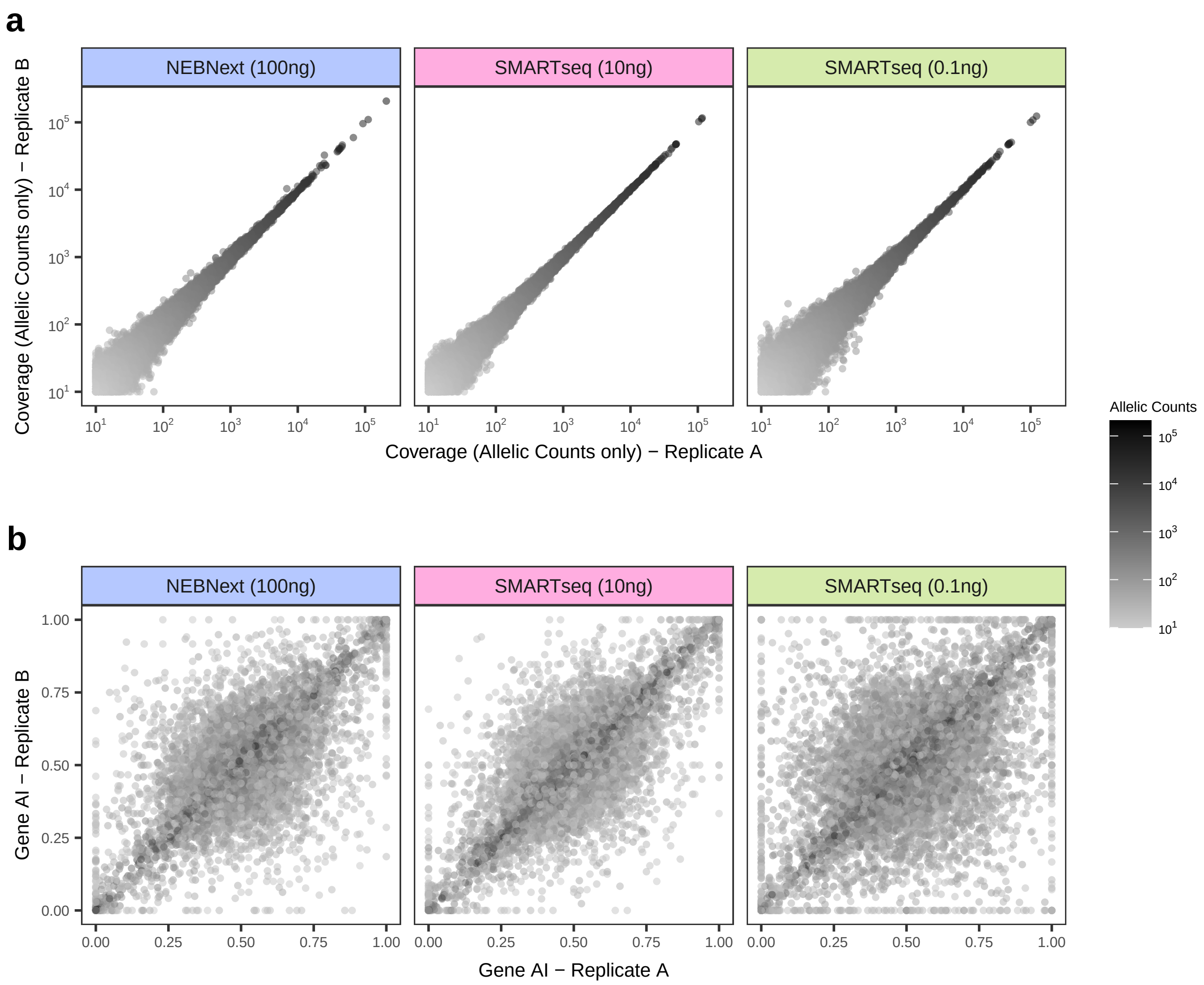

Figure S 2: **Comparison of agreement between technical replicates in coverage and in AI.**

Comparison of two replicate RNA-seq libraries prepared from the same RNA from 129xCastF1 mouse kidney From *left* to *right*: *Experiment 1 [NEBNext(100ng)]*, *Experiment 2 [SMARTseq(10ng)]*, and *Experiment 3 [SMARTseq(0.1ng)]*. Total allelic counts reflected in greyscale; only genes with total allelic counts > 10 are shown.
a: Comparison of gene coverages (Allelic counts only);
b: Comparison of AI values [(maternal allelic counts)/(total allelic counts)].

Supplementary Figure S 3. Effect of QCC correction on concordance between results of AI imbalance test.

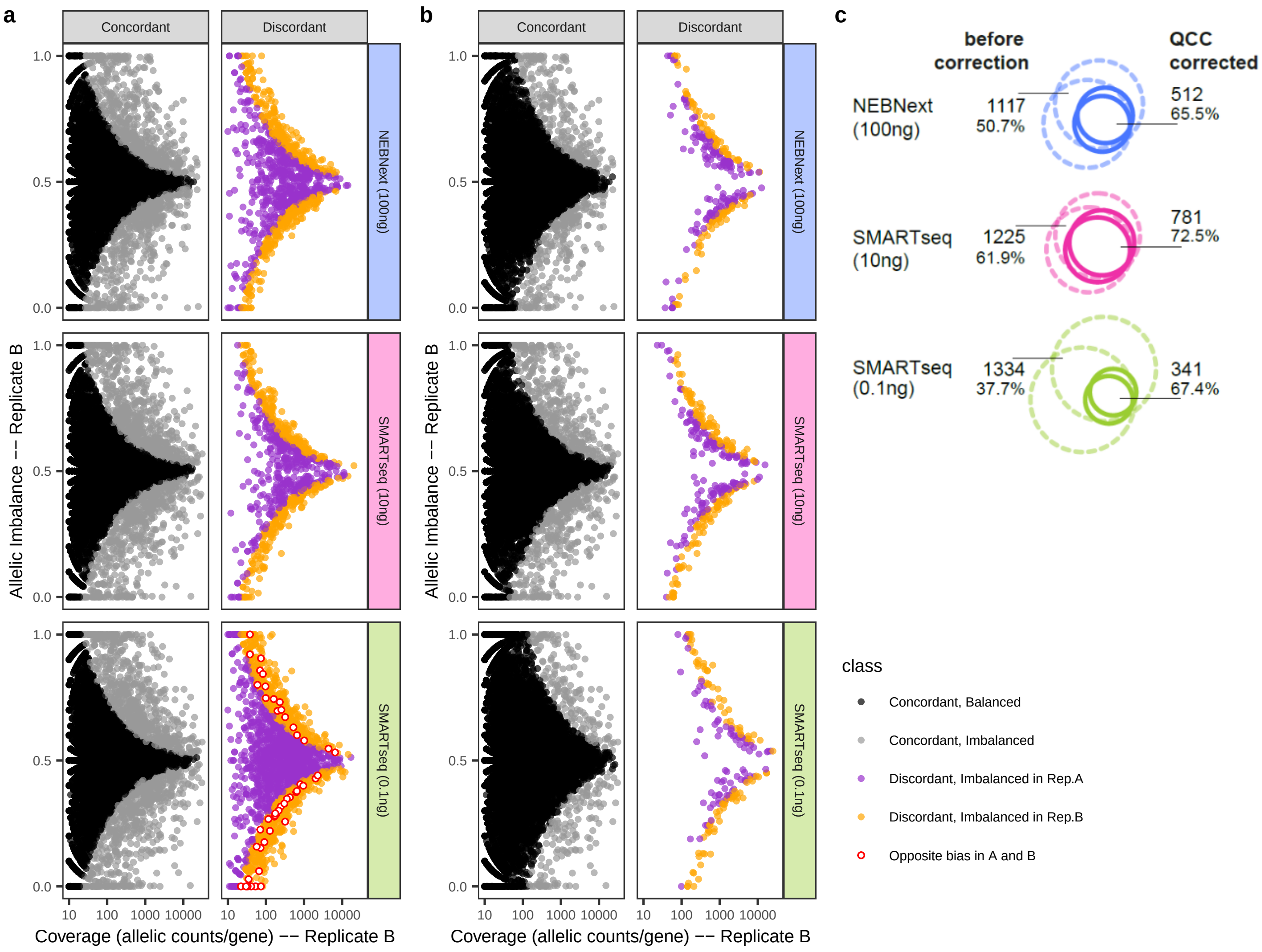

Figure S 3: **Effect of QCC correction on concordance between results of AI imbalance test.**

Tests applied are either binomial or QCC-corrected binomial (H_0: AI = 0.5 is rejected; p = 0.05 with Bonferroni correction). Only genes with total allelic coverage over 10 are assessed.

a-b: Maps of concordance (grey/black) and discordance (colors as described in legend; also see **Fig.2a**) between two technical replicates.

a: Binomial test. Discordance maps same as in **Fig.2b**.

b: QCC-corrected binomial test. Discordance maps same as in **Fig.2f**.

c: Concordance between genes classified as allelically imbalanced between pairs of RNA-seq replicate libraries (i.e., reflects all dots in panels **a,b** except black (concordant balanced)). Note that all 12 circles of this Euler diagram are scaled in proportion with each other. Dotted line: using binomial test; solid line: same replicates, using QCC-corrected binomial test. Top to bottom: *Experiment 1 [NEBNext (100ng)], Experiment 2 [SMARTseq (10ng)], Experiment 3 [SMARTseq (0.1ng)]*. Shown are absolute numbers of overlapping genes and % of the union constituted by the intersection (% of genes with concordant imbalance among genes called imbalanced at least in one replicate).

Supplementary Figure S 4. Concordance between replicates when using different tools for analyzing allele-specific expression.

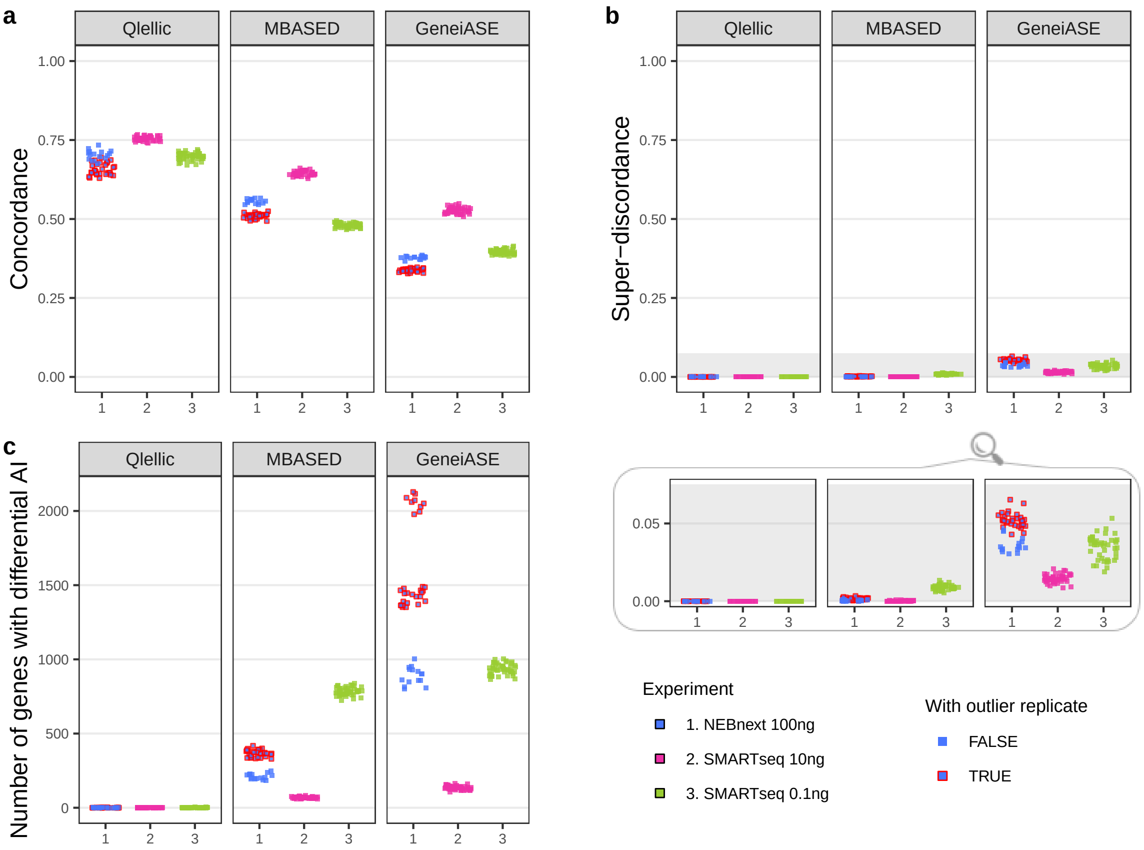

Figure S 4: **Concordance between replicates when using different tools for analyzing allele-specific expression.**

a: Concordance of lists of genes with significant AI (H_0: AI=0.5 rejected) between all pairs of combined replicates [see below for detailed explanation]. See also **Fig.2e**.
b: Proportion of "super-discordant" genes - such that the opposite significant bias is found in each side of the comparison. See also **Fig.2b,f,g**. Bottom: Zoom-in of the same plot.
c: Number of genes with significant differential AI (H_0: $\text{AI}_A = \text{AI}_B$ rejected) – note that all of these are from technical replicates and thus are by definition false positives. For MBASED, the union of its asymmetrical calls (A vs B gives a different set from B vs A) is taken. See also **Fig.4a**.
A unit of comparison here is some combination of two individual replicates (e.g., [30M reads from replicate 1 combined with 30M reads from replicate 2] compared to [30M reads from replicate 3 combined with 30M reads from replicate 4]). Shown are all 45 possible pairwise comparisons between such combinations.
Same reads and same genes were used as input for all tools. SNP count tables were created with ASEReadCounter* pipeline. For Qllelic, they were processed as described in this paper. For MBASED and GeneiASE, counts from the two replicates were summed up since these tools do not provide other utilisation of replicates.
Same sets of genes were used after filtering (in each of the 45 comparisons) using requirements and restrictions of all tools:
– after merging counts in replicate pair, gene should has less than 23 SNPs non-zero covered for each side of comparison, not necessarily the same (MBASED: fails to process genes with at least 23 SNPs covered)
– genes should have have coverage greater or equal than 1 in each replicate, and greater or equal than 8 in each joint replicate pair
a-c: Legend applies to the whole figure. All the combinations that include the outlier replicate #1 in NEBnext (100ng) experiment are framed in red.

Supplementary Figure S 5. Principal stages of RNA-seq experiment and data analysis.

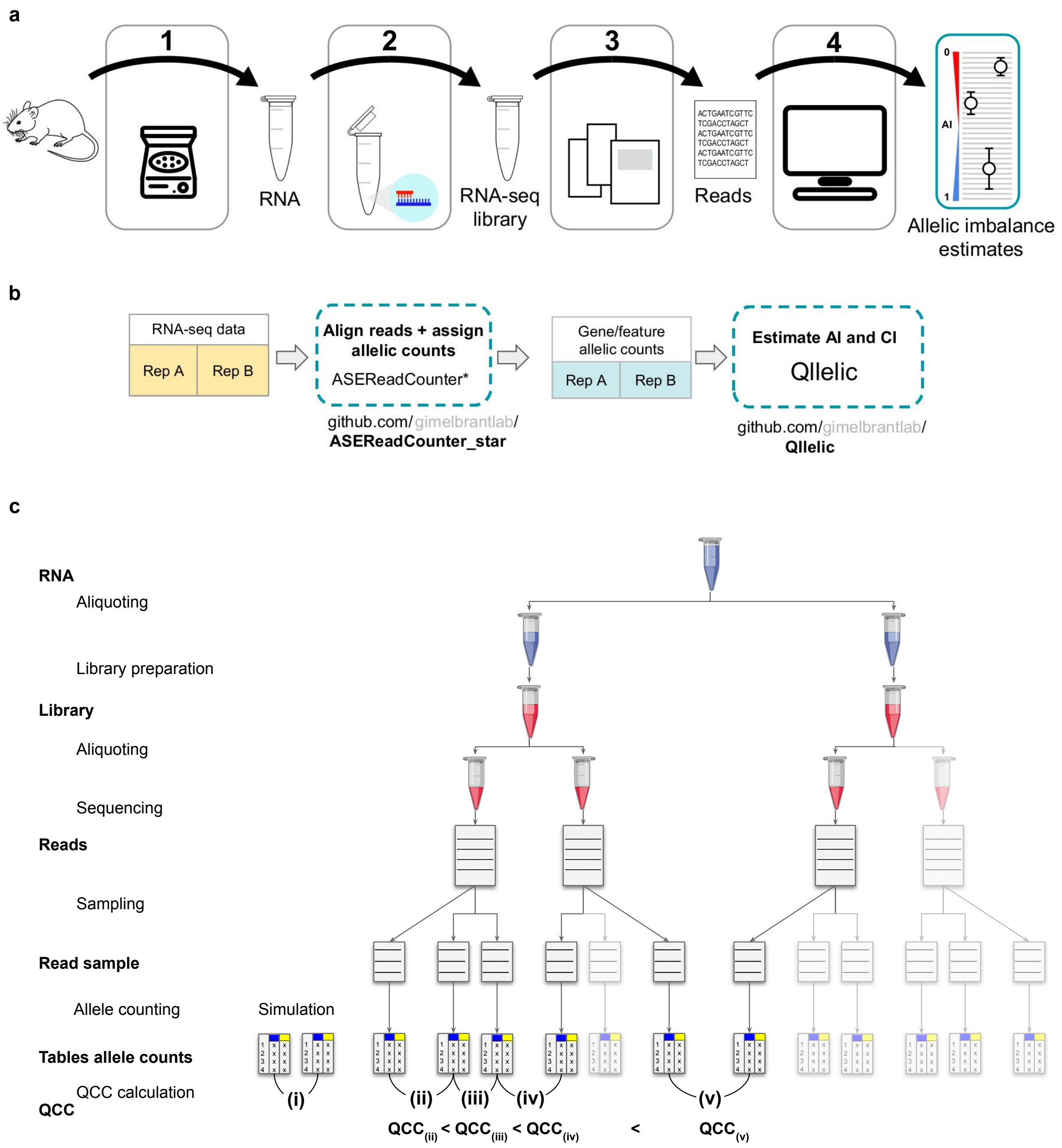

Figure S 5: **Principal stages of RNA-seq experiment and data analysis.**

a: Steps of RNA-seq experiment. 1 – RNA isolation; 2 – Library preparation; 3 – Sequencing, resulting in "reads" (which can be pairs in PE sequencing). Separate sequencing runs of the same library result in "physical subsampling" of the library; 4 – Data analysis.
b: Overview of data analysis with ASEReadCounter* and Qllelic. Note that other tools could be used for allele counting instead of ASEReadCounter* (e.g., see **Suppl. Fig. S6**).
c: How datasets were prepared for analysis of source of overdispersion in data processing. Comparisons (i) – (v) are described in detail in "Sources of AI overdispersion: data analysis" section of Results.

Adapted Wikicommons centrifuge image by user DataBase Center for Life Science (DBCLS); license: Creative Commons Attribution 4.0 International; Used Wikicommons mouse image by user Gwilz; license: Creative Commons Attribution-Share Alike 4.0 International.

Supplementary Figure S 6. Assignment of allele-uninformative reads to haplotypes leads to increase in AI overdispersion.

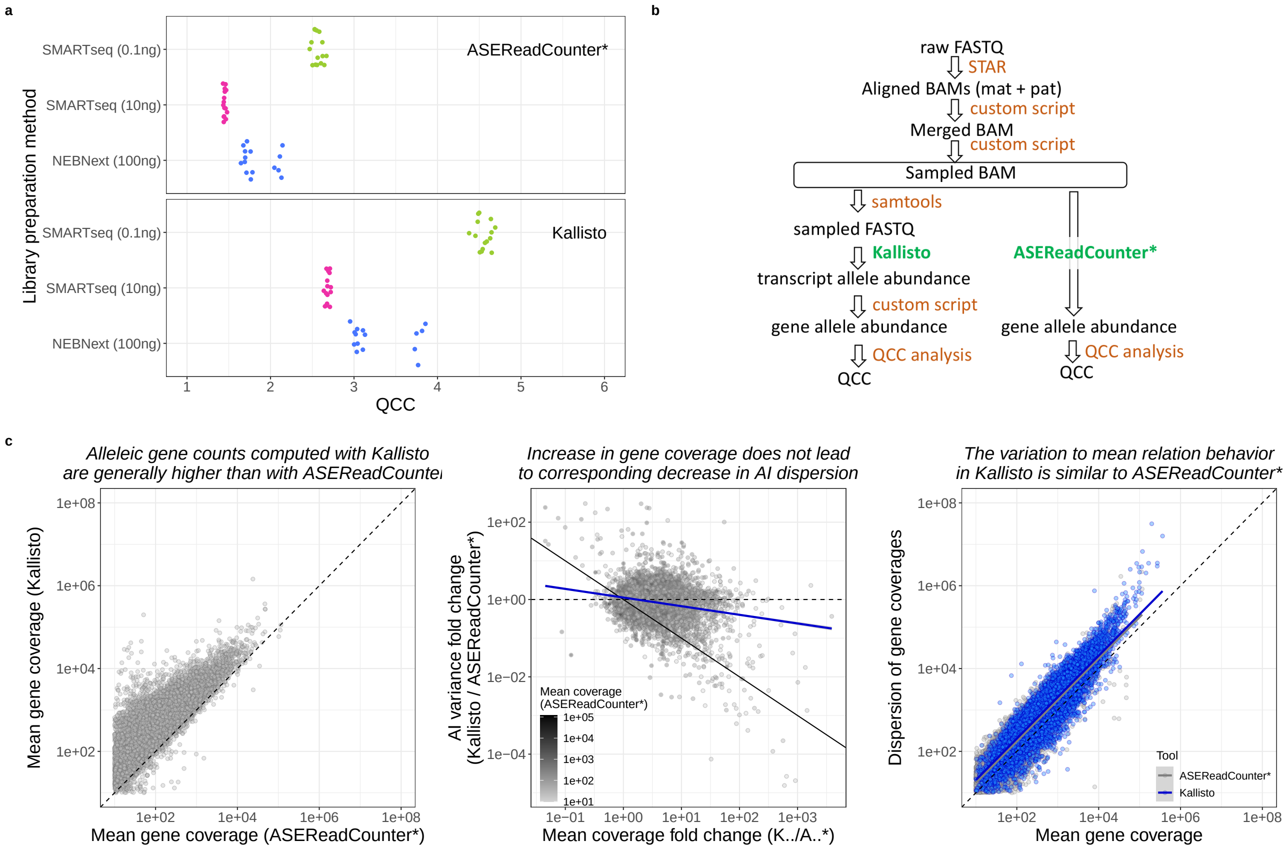

Figure S 6: **Assignment of allele-uninformative reads to haplotypes leads to increase in AI overdispersion.**

a: QCC values calculated for allelic counts computed from the same set of aligned reads by the default counter (ASEReadCounter*) and a tool that assigns allele-uninformative reads to haplotypes (Kallisto).
b: Outline of data processing with ASEReadCounter* and Kallisto. c: Source of elevated overdispersion when using Kallisto for allelic coverage counting.
Left - Kallisto pipeline yields higher allelic counts (due to assignment of non-SNP-covering reads to allelic counts).
Center - Despite higher allelic counts in Kallisto, no great decrease in AI variance (black solid line - expected relationship between fold change in AI variance and fold change in mean gene coverage change; dashed line - expected if AI variance were independent of mean coverage; blue line - linear fit of observed relationship).
Right - Overall relationship between gene coverage and AI dispersion is similar for Kallisto (blue) and ASEReadCounter* (grey), while Kallisto output has higher overdispersion. Also compare to **Fig.4e**.
For this figure, analyses performed on 30M reads sampled from each of six replicates in the SMARTseq (10 ng) dataset.

Supplementary Figure S 7. QCC captures simulated AI overdispersion.

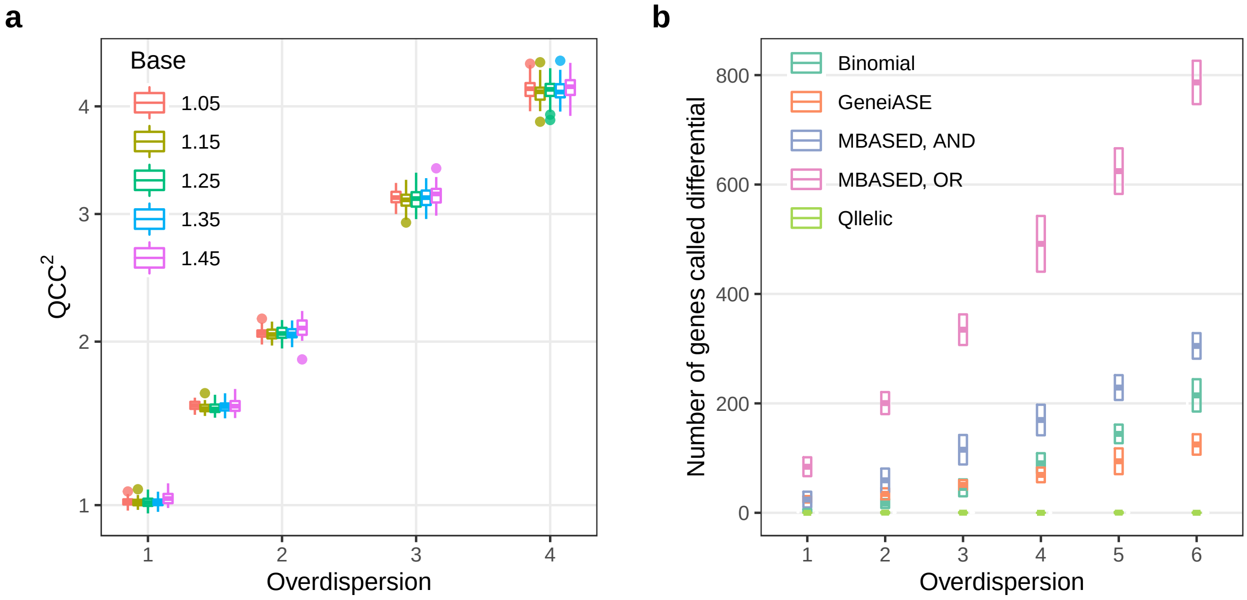

Figure S 7: **QCC captures simulated AI overdispersion.**

a: Gene coverage bin size doesn't substantially change QCC estimate. QCC values were calculated for simulated data with preset AI overdispersion values (horizontal axis), for a range of coverage bin sizes (color-coded by exponential bin base as noted). Note that calculated overdispersion values (QCC) are nearly perfectly correlated with overdispersion values set in the simulation.
b: Performance of Qllelic, MBASED, GeneiASE and binomial test on simulated replicate data with preset AI overdispersion: number of false positive calls, i.e. genes classified as having significant differential AI (rejected H_0: $\mathrm{AI}_A = \mathrm{AI}_B$) when comparing simulated technical replicates. Due to the asymmetry of the MBASED outputs (the resulting p-values depend on the order of input samples), the results for each replicate pair were either joined (OR) or intersected (AND) in this analysis. Note that predefined overdispersion wasn't provided to Qllelic; instead, QCC was estimated from the simulated data.
a,b: Boxplot elements – center line: median; box: upper and lower quartiles; whiskers: 1.5x interquartile range; points: outliers.

Description of simulations:

(a) Coverage values were taken from mean coverage from 30-mln NEBnext experiment data processed with ASEReadCounter* (from 30-mln data, 5 samples per each replicate, only genes with mean coverage >8 were taken, and the number of genes in this simulation was determined by this number, 11,278). True AI values were drawn from a mixture of symmetrical beta distributions with parameters $\alpha_1 = 40$ and $\alpha_2 = 1/3$, weighted 9:1 respectively. Then coverage and AI values were randomly paired and used for generation of simulated replicate data.
For fixed overdispersion rate (1, 1.5, 2, 3, 4), coverage "observations" for 10 simulated replicates were drawn from Poisson distribution with λ values as rounded fractions of coverage values and the overdispersion parameter. Then maternal coverage "observations" were generated for each simulated replicate and gene using binomial distribution, with respective coverage "observation" and true AI value as parameters. Maternal and paternal coverages were then restored via multiplication of AI and (1-AI) by overdispersion.

(b) *Simulated transcriptome:*
Lengths of genes: longest transcript lengths from mouse genes, taken from Ensembl annotation. MBASED requires as input identities of ref and alt alleles in SNPs. These were generated randomly. SNP positions were

randomly generated with probability of SNP at each site of 1/120.
Simulated true values:
Coverage, AI, and gene length values were extracted (for 22,263 genes with non-zero coverage) from 30-mln SMARTseq 10ng experiment data as computed by Kallisto. Calculation of the average values from 6 replicates was performed using Qllelic functions `MeanCoverage()` and `CountsToAI()`.
Simulated allelic gene coverage:
Overdispersion: from 1 to 6. Total gene coverage “observations” for 10 simulated replicates were modeled with Poisson distribution, with λ values taken from coverage estimates. Maternal counts were then simulated with binomial distribution, using respective AI true proportion and a coverage “observation” divided by overdispersion, then multiplied by the overdispersion value and rounded. Paternal counts were obtained via subtraction of maternal counts from total counts.
Simulated allelic SNP counts and gene counts:
Read start positions were randomly picked among possible sites, in an amount determined by simulated allelic gene counts in the previous step. For each SNP the allelic coverage was counted as a number of reads covering the site. For Qllelic analysis, gene counts were taken as a sum of SNP counts on genes.
For further comparison, data was filtered as described in **Suppl. Fig. S4**, which left 6069 ± 12.2 genes. The pairs of replicates used for comparison were as follows: 1&2, 3&4, 5&6, 7&8 and 9&10 (10 combinations of 2+2 replicates overall).

Supplementary Figure S 8. Sources of AI overdispersion: impact of in-silico sampling and repeated sequencing runs (physical library sampling).

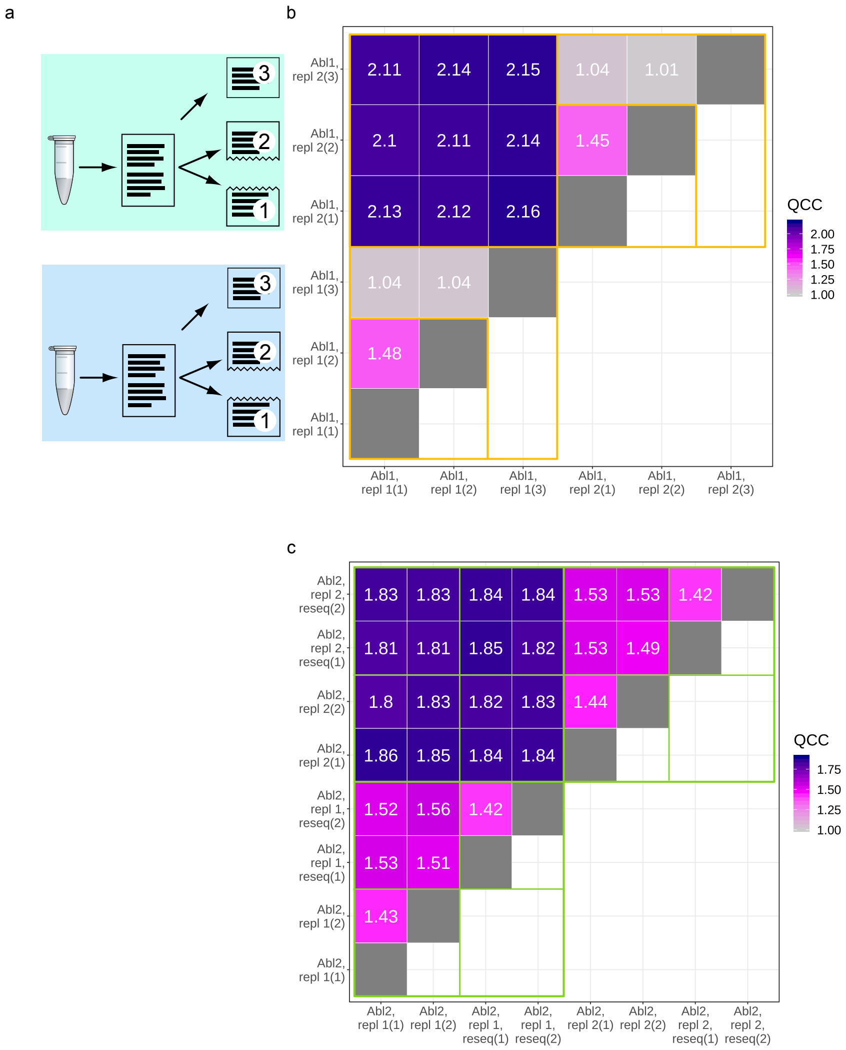

Figure S 8: **Sources of AI overdispersion: impact of in-silico sampling and repeated sequencing runs (physical library sampling).**

a: Schematics of the sampling. "1" and "2" are two non-intersecting subsets of reads [see definition in **Suppl. Fig. S4c**] from the same sequencing run of a particular replicate after alignment and assignment to parental genomes (paired reads were kept together). "3" is an independent sampling (of the same size as "1" and "2") of the same pool of reads. It is in binomial relationship with each of "1" and "2", e.g., it may overlap with these subsets.
b: For within-replicate comparison, in-silico sampling without return has greater contribution to AI overdispersion than binomial sampling. Pairwise QCC analysis performed on Abl.1 data, replicates 1 and 2. Subsets "1" and "2" are non-intersecting subsets (15,136,606 fragments each) of a given library sequencing run; "3" random subset of 15,136,606 fragments from the same sequencing run. Orange boxes highlight comparisons between "1" and "2" and between them and "3".
c: Repeated sequencing runs of the same library ("physical sampling") has small impact on AI overdispersion. Pairwise QCC analysis on Abl.2 data, replicates 1 and 2. Resequencing as annotated. Green boxes highlight comparisons within and between resequencing runs.

Supplementary Figure S 9. Sources of AI overdispersion: impact of deduplication.

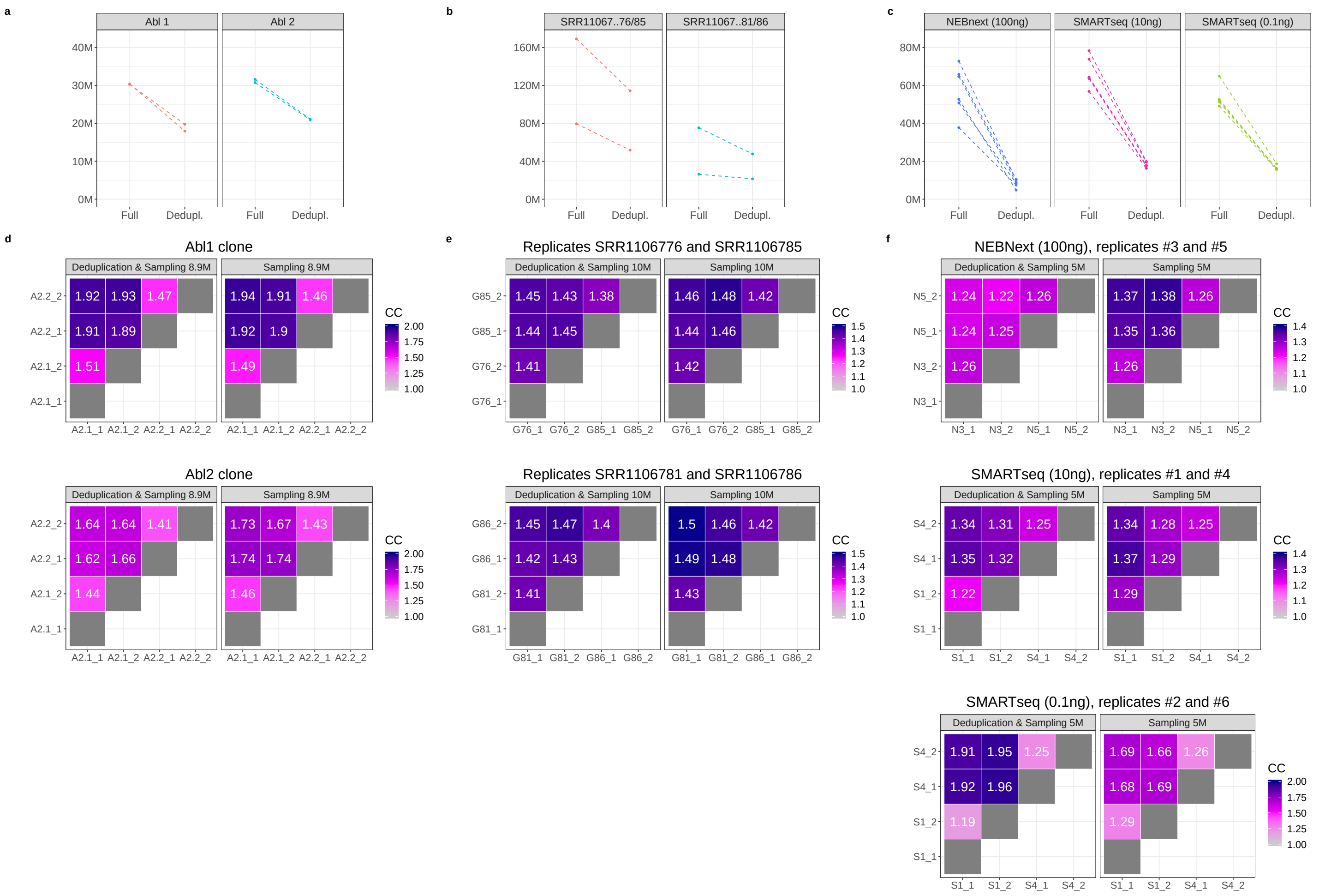

Figure S 9: **Sources of AI overdispersion: impact of deduplication.**

a, b, c: Number of fragments remaining after deduplication (using Picard MarkDuplicates).
a - PE150 data from Abl.1 and Abl.2
b - PE75 data from NPC cells (Gendrel dataset)
c - SE75 data for the kidney RNA-seq data in Experiments 1-3.
d, e, f: QCC after deduplication is still higher for comparing two replicates than comparing halves of one replicate.
d - QCC before and after deduplication: Abl.1 and Abl.2 data
e - QCC before and after deduplication: NPC cells (Gendrel dataset)
f - QCC before and after deduplication for randomly chosen pairs of replicates of kidney RNA-seq data. Note that QCC can become higher after deduplication.

Supplementary Figure S 10. Goodness of fit - R^2 for observed and expected quantiles.

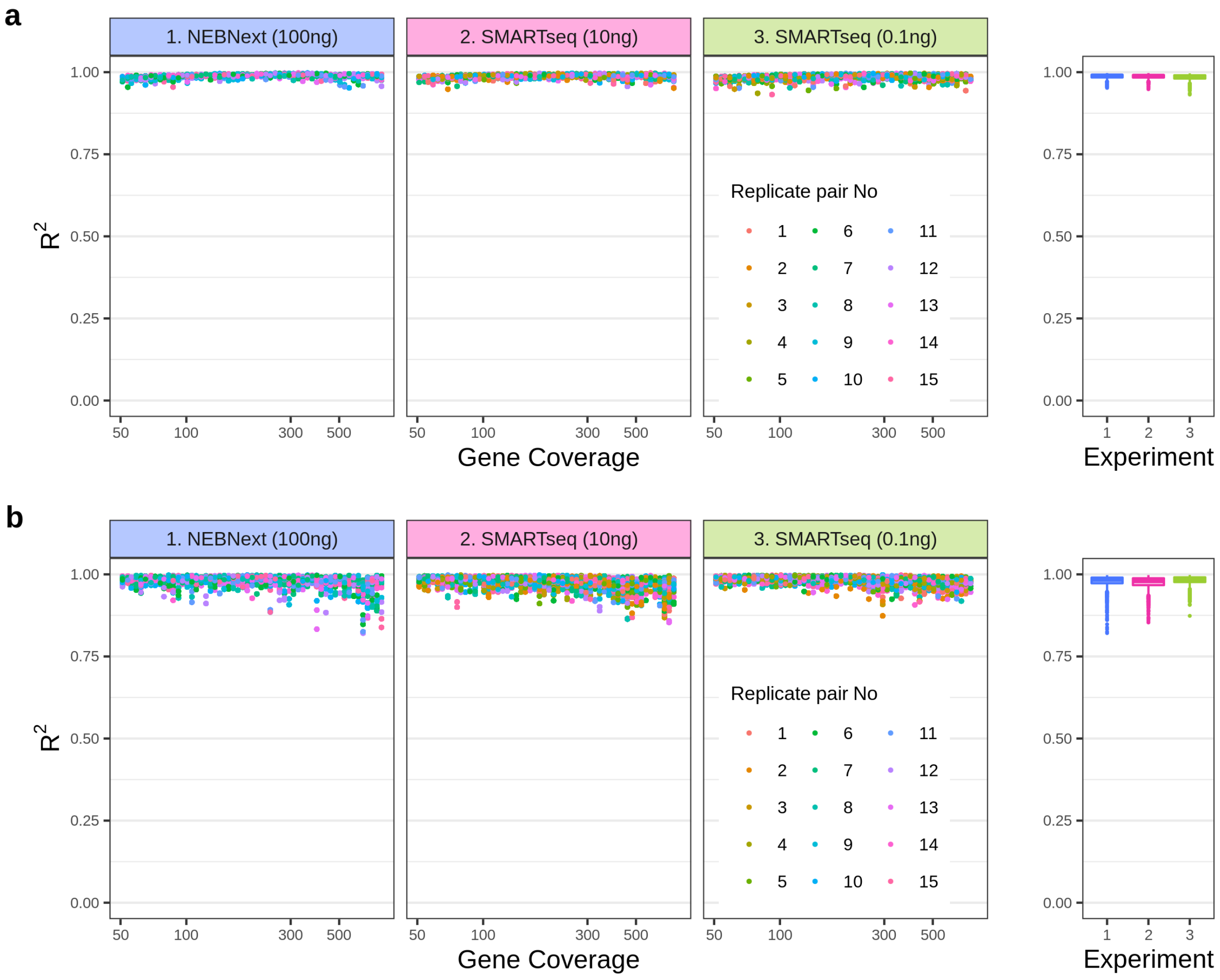

Figure S 10: **Goodness of fit - R^2 for observed and expected quantiles.**

R^2 statistic was computed for linear regression between observed and expected quantile values (from 0.025 to 0.975 with step 0.025), using the `summary.lm` function from the `stats` R package. Each point indicates a coverage bin and one of the 15 pairs of replicates analysed together (the outlier replicate in Experiment 1 was removed from this analysis).
a: Goodness of QCC fit with a linear regression model, on quantile values for observed and modeled ΔAI distribution (for more information see **Fig.3f**).
b: Goodness of AI fit with the Beta-Binomial mixture model, on quantile values for observed and modeled AI distribution (for more information see **Fig.3d**). For each pair of replicates, comparison of simulated replicates was performed with both actual replicates.
a,b: Boxplot elements – center line: median; box: upper and lower quartiles; whiskers: 1.5x interquartile range; points: outliers.

Supplementary Figure S 11. Goodness of fit - QQ plots.

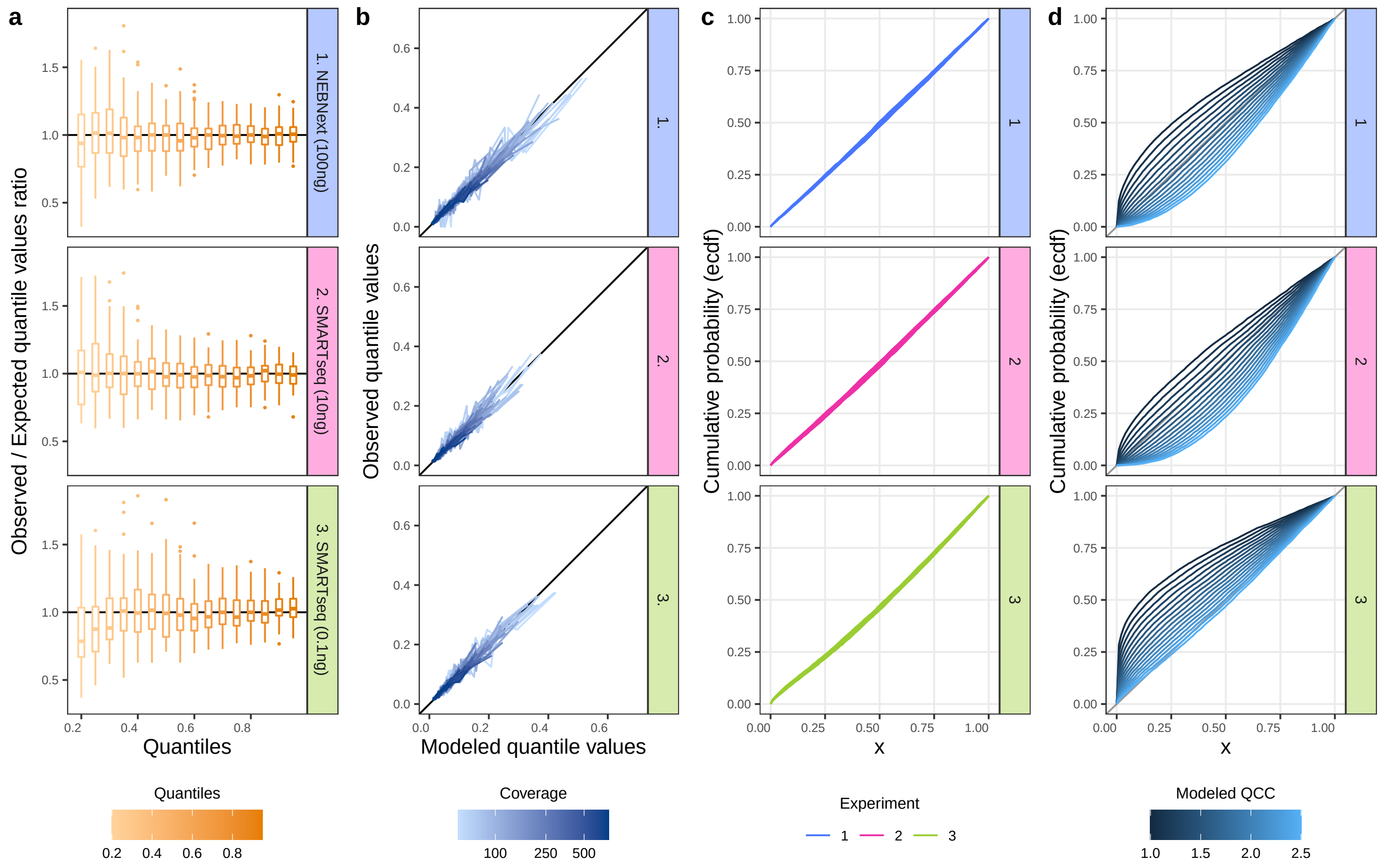

Figure S 11: **Goodness of fit - QQ plots.**

a, b: After computing QCC, AI estimates for two simulated replicates (see **Fig.3d**) were re-generated using fitted Beta-parameters for AI distribution and introducing additional noise to binomial sampling according to QCC (gene coverage QCC^2 fold smaller). Quantile values (from 0.20 to 0.95 with step 0.05) were then compared for ΔAI distributions of modeled and observed data.
a: Boxplots of proportions of quantile values between observed and modeled data. Note that these values are distributed around 1, suggesting that the corrected model fits data well. Each boxplot shows quantile proportions for different coverage bins, for an example pair of replicates (rep. 3 vs 4 in each experiment).
b: Quantile-quantile plots for observed and modeled distributions, for an example pair of replicates (rep. 3 vs 4 in each experiment). Coverage bin is depicted with color.
c: Empirical cumulative distribution function (ecdf) for p-values of differential test between all 45 sets of two replicate pairs within each experiment (15 sets in NEBnext experiment, since the outlier rep. 1 was excluded), performed with respective calculated QCCs. Note that it is close to the x=y diagonal, as expected for an appropriate test.
d: Empirical cumulative distribution function for p-values of differential test between two pairs of replicates (2&3) vs (4&5), for modeled QCC from 1 to 2.5 (with step 0.1). The curves *below* diagonal represent overly conservative tests, when curves *above* diagonal reflect underestimated dispersion. Note that line close to the diagonal corresponds to QCC value as determined in Qllelic analysis (1.67 and 1.72 for the pairs in Expt.1; 1.45 and 1.47 for Expt.2; 2.62 and 2.53 for Expt.3).

Supplementary Figure S 12. Goodness of fit - at a single-gene level, AI standard deviation meets expectations.

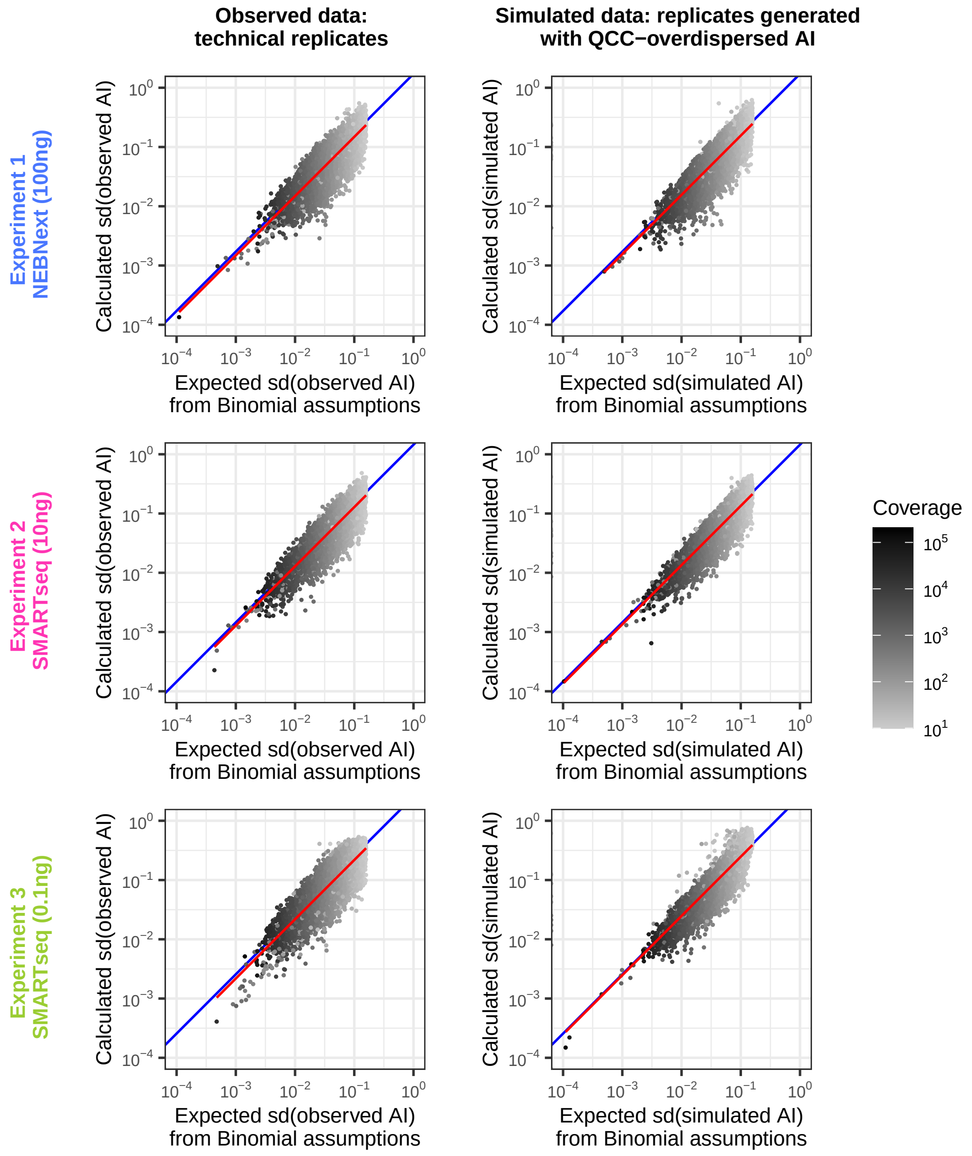

Figure S 12: **Goodness of fit - at a single-gene level, AI standard deviation meets expectations.**

Regression between calculated sd(AI) and expected sd(AI) for gene-level data (red line) follows the QCC trend (blue line) in both real data (*left*) and simulated data (*right*). *Top* to *bottom* - experiments 1 through 3; AI values and coverages from respective real data were used for simulations.

Calculated sd(AI) values were computed on 5 replicates (Expt.1, with outlier replicate removed) or all 6 replicates for Expts. 2 and 3.

Expected sd(AI) values were estimated from binomial expectations: $\sqrt{\frac{\text{AI}\cdot(1-\text{AI})}{\text{coverage}}}$. AI point estimates and coverage were from all pooled replicates.

For simulations, mean observed coverage values were taken as λ parameter for Poisson distribution, used to simulate coverage for the number of replicates in each experiment (i.e. 5 or 6). AI values were taken from observed point estimates, and binomial distributions on counts divided by experimentally determined QCC^2 were used to generate maternal counts.

Supplementary Figure S 13. Expectation-maximization fitting of allelic imbalance with Beta-Binomial mixture distribution.

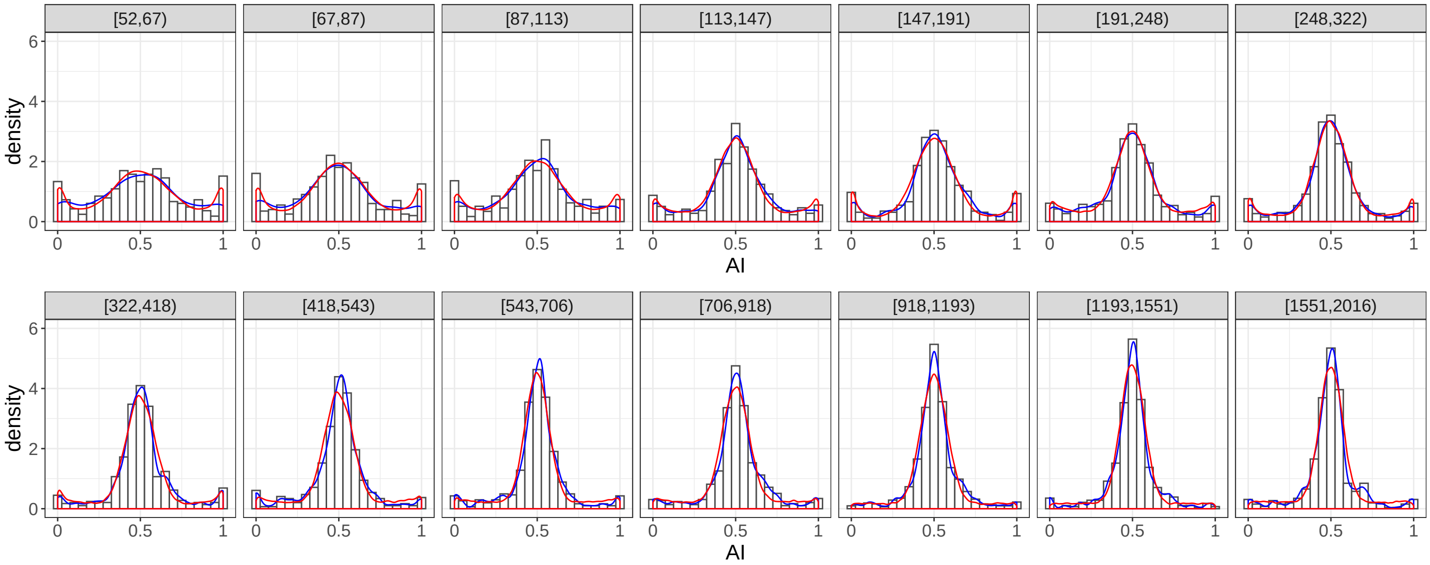

Figure S 13: **Expectation-maximization fitting of allelic imbalance with Beta-Binomial mixture distribution.**

Observed AI distribution in the actual data in gene coverage bins (as annotated in each box). Note that the smoothed density plot of the histogram (blue line) is closely matched with fitted distribution (red line).
Data: non-deduplicated RNA-seq for Abl.1 clone, sampled to 30,273,212 PE150 reads in each of two technical replicates; base of exponential binning is 1.3.

Supplementary Figure S 14. Relation between QCC and accounting for AI overdispersion using Beta-Binomial distribution with sample-specific overdispersion parameter ρ.

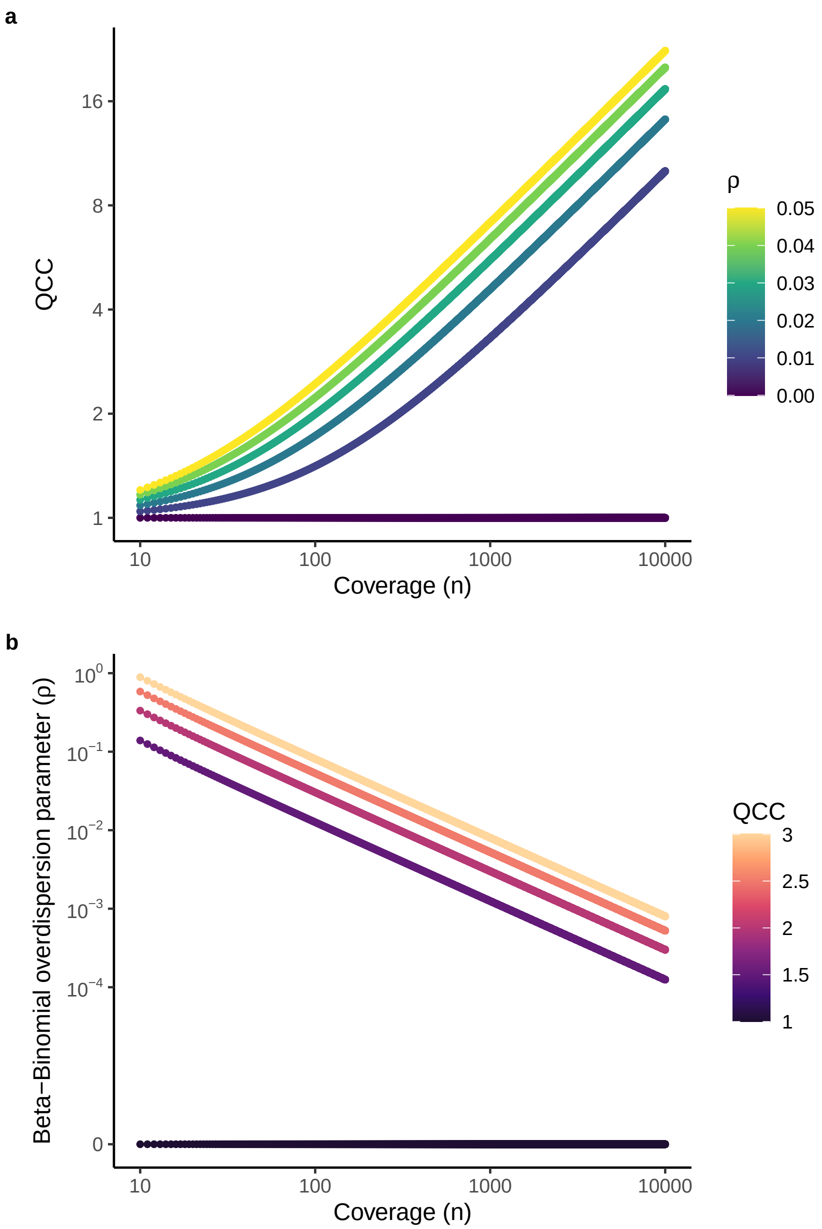

Figure S 14: **Relation between QCC and accounting for AI overdispersion using Beta-Binomial distribution with sample-specific overdispersion parameter ρ.**

a: QCC value that corresponds to a given parameter ρ, that provides the same width of AI distribution at the particular coverage.
b: Overdispersion parameter ρ that corresponds to a given QCC value, that provides the same width of AI distribution at the particular coverage.
Note that using $\rho = 0.03$ (Mayba et al., 2014) would lead to underestimation of AI overdispersion at low covered genes and overestimation of AI overdispersion at highly covered genes.

Supplementary Figure S 15. AI overdispersion remains experiment-specific when calculated for individual SNPs.

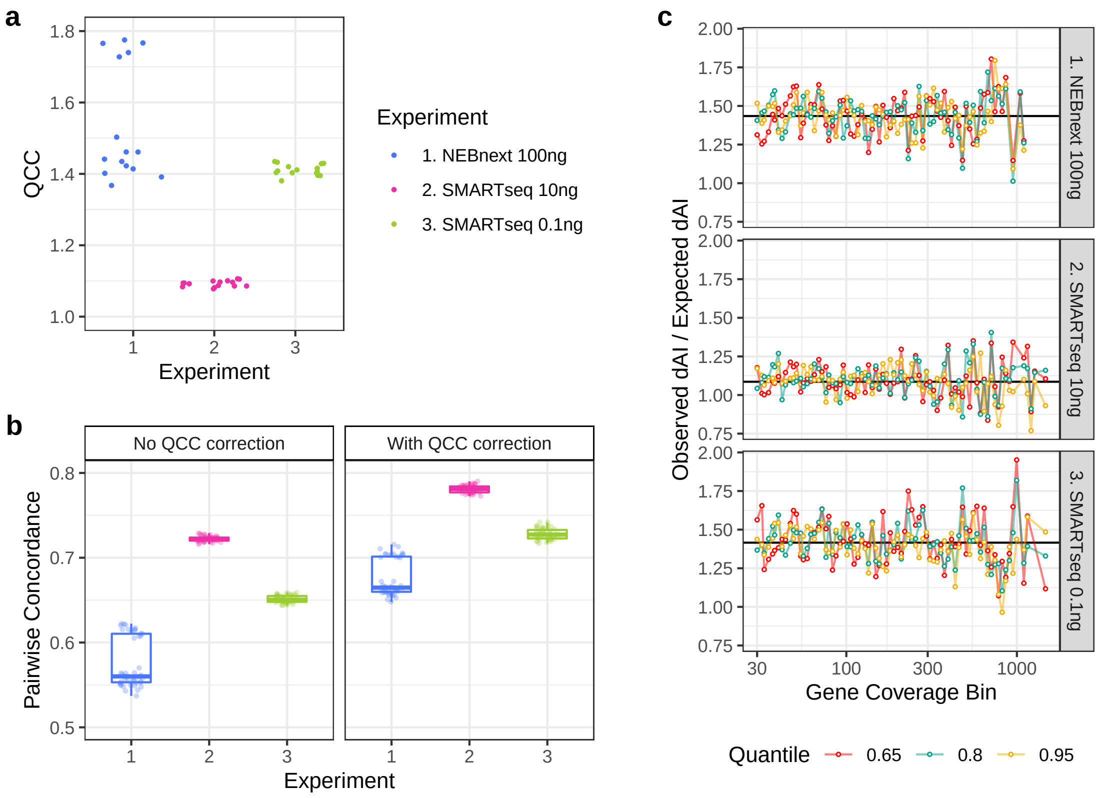

Figure S 15: **AI overdispersion remains experiment-specific when calculated for individual SNPs.**

a: QCC values computed for all possible pairs of replicates for all three experiments (compare to **Fig.2d**).
b: Concordance of "allelically imbalanced" genes in all possible pairs of replicates, H_0 : AI = 0.5 is rejected by binomial (*left*) or QCC-corrected binomial test (*right*) test; confidence level is 0.95, with Bonferroni correction (compare to **Fig.2e**). Boxplot elements – center line: median; box: upper and lower quartiles; whiskers: 1.5x interquartile range; points: outliers.
c: Ratios of observed and expected values of ΔAI for three example quantiles across coverage bins for two example replicates taken from each of the three experiment (compare to **Fig.3f**)

Supplementary Figure S 16. Impact of QCC value on analysis of allele-specific expression in an example GTEx dataset.

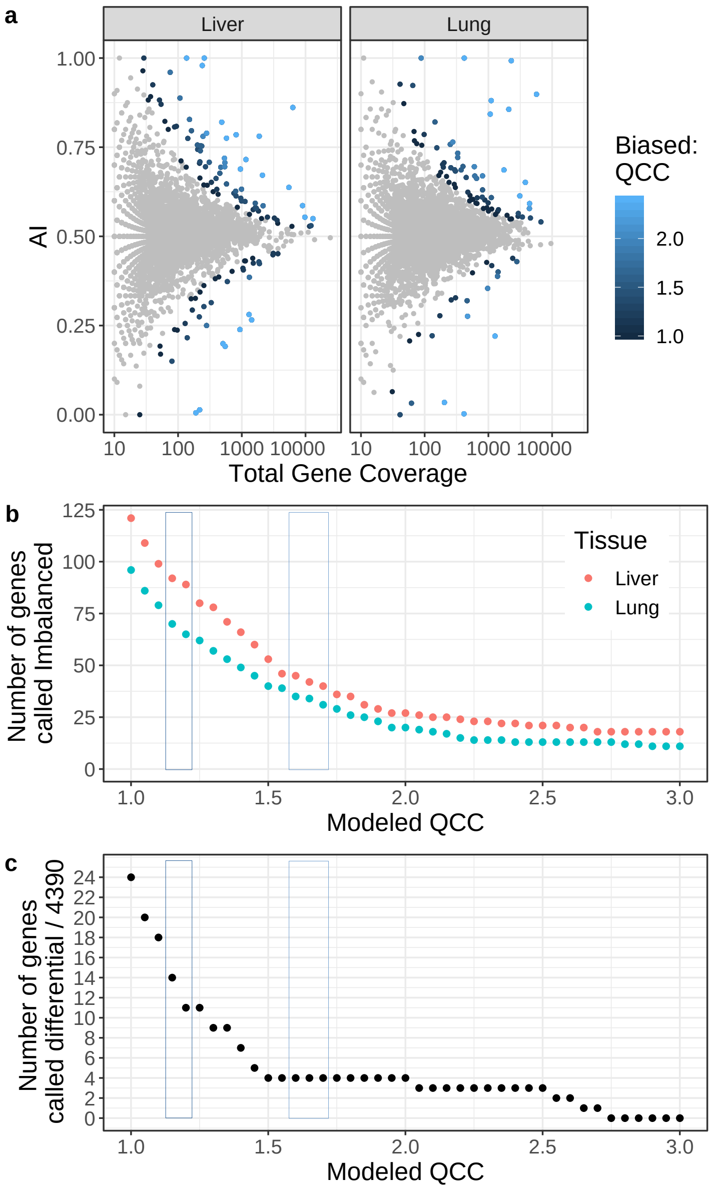

Figure S 16: **Impact of QCC value on analysis of allele-specific expression in an example GTEx dataset.**

To illustrate the impact of overdispersion on the analysis of expression AI in a dataset with no replicates, we assessed RNA-seq data for two human tissues, liver and lung, from the same arbitrarily selected individual (GTEX-11NUK) from the GTEx project (no replicates available). Allelic counts for these samples are provided in the Source Data provided with this paper (`SourceData.zip` file).

a: Distribution of significantly biased genes (testing $H_0 : \mathrm{AI} = 0.5$) at different assessed QCC values from 1.0 (no overdispersion) to 3.0.

b: Same analysis as in **a**, showing the number of imbalanced genes for the two tissues.

c: Differential analysis of expression AI between the two tissues, when corrected using different QCC values (equal for both samples).

Colored boxes correspond to the range of QCCs observed in the Geuvadis data (1.15-1.2) and our data from the NEBnext experiment (1.6-1.7).

. Supplementary Tables .

for Mendelevich et al., *"Replicate sequencing libraries are important for quantification of allelic imbalance"*

Contents

Supplementary Table S 1. RNA-seq datasets analyzed in this study 21
Supplementary Table S 2. Analysis of technical replicates of RNA-seq from human cell lines 22
Supplementary Table S 3. Overdispersion and other features for RNA-seq from mouse neuronal progenitor cells 23

Supplementary Table S 1. RNA-seq datasets analyzed in this study

Sample	Replicates	# fragments	Seq type	GEO ID	Reference
F1 129S1/CAST kidney, Exp 1 (NEBnext 100ng)	1	63850091	SE 75	GSE143310	this work
	2	77092011			
	3	87531947			
	4	45547354			
	5	79260529			
	6	61713808			
F1 129S1/CAST kidney, Exp 2 (SMARTseq 10ng)	1	85084237	SE 75		
	2	73162672			
	3	65565213			
	4	74019699			
	5	73733135			
	6	90229986			
F1 129S1/CAST kidney, Exp 3 (SMARTseq 0.1 ng)	1	57996564	SE 75		
	2	58816137			
	3	72585208			
	4	57195273			
	5	54874918			
	6	57807668			
mouse clone Abl.1	1	34228003	PE 150		
	2	33297046			
mouse clone Abl.2	1	33116795	PE 150		
	2	33929680			
mouse clone Abl.2 second round of seq	1	37381472	PE 150		
	2	36658803			
**Data from other studies**					
**Human RNA-seq data**					
Geuvadis study – see Supplementary Table 2					Lappalainen et al, (2013) Nature, 501(7468), 506-511.
GTEx study:					GTEx Consortium, (2015) Science, 348(6235), 648-660.
liver	GTEX-11NUK-1226-SM-5P9GM				
lung	GTEX-11NUK-0826-SM-5HL4U				
**Mouse RNA-seq data**					
Neuronal progenitor cells (GSE54016) – see Supplementary Table 3					Gendrel et al, (2014) Dev Cell, 28(4), 366-380.

Supplementary Table S 2. Analysis of technical replicates of RNA-seq from human cell lines

Technical replicates were available for five samples in the Geuvadis project [Lappalainen, T., Sammeth, M., Friedländer, M. et al. Nature 501, 506–511 (2013) doi:10.1038/nature12531], with seven libraries generated from one RNA prep per each sample. Overdispersion and other AI metrics are shown for all pairwise comparisons within each replicate set (figures in bold show average and s.d.).

sample	technical replicates	# alligned fragments in sample (PE 75)	# genes w coverage >8	number of genes AI != 0.5 (assuming QCC = 1)	QCC value	number of genes AI != 0.5 (with QCC) in pairwise comparisons
HG00117	ERR205004	24,185,758	4609	269, 272, 272,	1.104, 1.124, 1.104,	225, 236, 236,
	ERR204894	29,013,132	4489	268, 275, 263,	1.152, 1.133, 1.113,	219, 225, 225,
	ERR204909	22,442,567	4617	285, 264, 277,	1.119, 1.093, 1.108,	234, 235, 228,
	ERR204950	28,202,246	4651	266, 269, 276,	1.096, 1.14, 1.148,	237, 216, 230,
	ERR204975	15,440,366	4351	269, 278, 272,	1.163, 1.172, 1.132,	216, 225, 224,
	ERR205006	26,668,943	4478	265, 273, 272,	1.138, 1.143, 1.155,	213, 227, 210,
	ERR204879	21,722,554	4605	280, 262, 271	1.124, 1.152, 1.162	226, 211, 214
			**4542.9 ± 107.51**	**271.3 ± 5.85**	**1.13 ± 0.023**	**224.4 ± 8.61**
HG00355	ERR204824	26,593,833	4793	212, 203, 205,	1.093, 1.109, 1.082,	189, 160, 180,
	ERR204831	28,088,219	4755	200, 199, 208,	1.088, 1.106, 1.173,	184, 170, 156,
	ERR204846	28,142,959	4737	204, 205, 194,	1.126, 1.13, 1.101,	167, 172, 173,
	ERR204854	24,273,505	4756	206, 205, 199,	1.13, 1.142, 1.115,	172, 175, 158,
	ERR204901	23,717,053	4760	195, 196, 202,	1.088, 1.148, 1.169,	172, 155, 150,
	ERR204953	29,204,264	4726	204, 201, 203,	1.076, 1.126, 1.163,	187, 160, 164,
	ERR204972	18,434,828	4503	200, 205, 198	1.09, 1.144, 1.137	171, 166, 170
			**4718.6 ± 97.34**	**202.0 ± 4.42**	**1.12 ± 0.029**	**169.1 ± 10.49**
NA06986	ERR204855	30,501,691	4654	295, 300, 304,	1.125, 1.143, 1.154,	250, 255, 236,
	ERR204860	22,698,694	4633	310, 308, 315,	1.141, 1.112, 1.083,	241, 261, 267,
	ERR204863	27,287,384	4546	301, 299, 298,	1.113, 1.158, 1.165,	257, 230, 232,
	ERR204929	18,328,969	4494	308, 320, 313,	1.107, 1.108, 1.101,	259, 272, 266,
	ERR204955	24,106,890	4616	307, 322, 316,	1.129, 1.058, 1.045,	253, 297, 290,
	ERR204968	23,724,945	4641	304, 322, 313,	1.161, 1.142, 1.104,	241, 256, 264,
	ERR205005	23,033,870	4631	316, 310, 332	1.148, 1.121, 1.098	243, 265, 280
			**4602.1 ± 59.19**	**310.1 ± 9.35**	**1.12 ± 0.032**	**257.9 ± 17.77**
NA19095	ERR204843	28,887,221	5782	267, 278, 255,	1.157, 1.159, 1.208,	211, 211, 196,
	ERR204858	28,260,083	5991	262, 258, 274,	1.151, 1.185, 1.192,	204, 200, 183,
	ERR204861	18,124,458	5719	255, 262, 263,	1.098, 1.094, 1.099,	219, 226, 224,
	ERR204868	29,178,865	5901	256, 271, 261,	1.111, 1.077, 1.117,	223, 234, 217,
	ERR204891	22,350,859	5984	262, 246, 253,	1.102, 1.087, 1.085,	218, 214, 225,
	ERR204930	19,336,064	6018	264, 258, 271,	1.112, 1.118, 1.122,	219, 213, 214,
	ERR205009	25,050333	5965	255, 279, 255	1.142, 1.103, 1.125	201, 228, 215
			**5908.6 ± 115.22**	**262.1 ± 8.61**	**1.13 ± 0.037**	**214.0 ± 12.01**
NA20527	ERR204830	32,519,775	4766	213, 211, 209,	1.097, 1.063, 1.077,	184, 189, 184,
	ERR204874	25,882,966	4849	220, 204, 206,	1.118, 1.15, 1.068,	178, 166, 188,
	ERR204908*	12,598,312	4728	208, 212, 211,	1.11, 1.11, 1.141,	178, 187, 168,
	ERR204934	24,903,624	4825	204, 208, 213,	1.079, 1.112, 1.123,	190, 183, 187,
	ERR204965	19,808,430	4862	222, 210, 209,	1.176, 1.132, 1.133,	168, 173, 176,
	ERR204978	33,101,465	4836	205, 207, 219,	1.12, 1.188, 1.107,	182, 159, 186,
	ERR204993	27,981,881	4855	205, 212, 201	1.177, 1.107, 1.095	155, 179, 178
			**4817.29 ± 50.73**	**210.0 ± 5.44**	**1.12 ± 0.034**	**178.0 ± 9.92**

* - this sample had the fewest reads; to ensure uniform analysis, all the sampling was performed to this depth.

Supplementary Table S 3. Overdispersion and other features for RNA-seq from mouse neuronal progenitor cells

2 biological replicates were available for 2 samples [Gendrel, A.V. et al. Dev Cell. 2014 Feb 24;28(4):366-80 doi: 10.1016/j.devcel.2014.01.016]. Overdispersion and other AI metrics are shown for a replicate pair.

sample	biological replicates	# alligned fragments in sample (PE 100)	# genes w coverage >8	number of genes AI != 0.5 (with QCC = 1)	QCC value	number of genes AI != 0.5 (with QCC) in pairwise comparisons
SRS529152<br>SRS529162	SRR1106776<br>SRR1106785	79,524,208<br>169,065,784	12531<br>12661	3338	1.51	1995
SRS529159<br>SRS529163	SRR1106781<br>SRR1106786*	75,319,044<br>26,302,221	12383<br>12373	3104	1.56	1699

* - this sample had the fewest reads; to ensure uniform analysis, all the sampling was performed to this depth.

. Supplementary Notes .

for Mendelevich et al., *"Replicate sequencing libraries are important for quantification of allelic imbalance"*

Contents

Supplementary Note S 1. Is one technical replicate sufficient to separate AI signal from noise? 25

Supplementary Note S 2. Accounting for overdispersion leads to the expected bimodal distribution of AI values in discordant AI calls. 30

Supplementary Note S 3. Genes with different underlying AI have different impact on the overall signal variance. 33

Supplementary Note S 4. We expect nearly zero genes with false positive AI, when we estimate AI and CI from two replicates and then calculate AI from six replicates. 34

Supplementary Note S 5. Statistical power of the QCC-corrected test. 36

Supplementary Note S 6. Worked example of QCC calculation, starting from fastq. 39

Supplementary Note S 7. Worked example of AI differential analysis for two samples. 39

Supplementary Note S 1. Is one technical replicate sufficient to separate AI signal from noise?

A single technical replicate could provide sufficient data to separate AI signal from noise in some particular circumstances.

One such situation is if we know the exact distribution of noise. E.g., if noise were due solely to the sampling process, then any contribution of noise to the observed signal can be approximated by a universal binomial model depending only on coverage and allelic proportion. However, it is well appreciated that there exists extra-binomial dispersion which is non-trivial to fit [1, 2, 3, 4].

Conversely, there are specific true signal distribution classes that, if the data behaved in that way, would allow deconvolution of the AI signal from observations based on a single technical replicate. An example of such hypothetical behavior of data is a distribution with 3 states of true AI values: biallelic 50:50 and complete biases 100:0 and 0:100 (see section 2 of this Note for more details).

However, apart from such extreme cases, even when the distribution classes for both true signal and noise are known, this does not guarantee the ability to distinguish between different parametrizations of signal and noise distributions. Here, we will consider several such cases.

1.1. Case of normally distributed signal and noise

To demonstrate the concept of the indistinguishable parameters in the distributions, let us take a variable $\bar{x} \sim \mathcal{N}(\bar{\mu}, \bar{\sigma}^2)$, and let measurement error be also normally distributed: $\varepsilon \sim \mathcal{N}(0, \sigma_\varepsilon^2)$. Then

$$\bar{x} \sim \mathcal{N}(\bar{\mu}, \bar{\sigma}^2)$$
$$x \sim \mathcal{N}(\bar{x}, \sigma_\varepsilon^2) \Leftrightarrow x \sim \mathcal{N}(\bar{\mu},\ \bar{\sigma}^2 + \sigma_\varepsilon^2)$$

Given a sample, the expected value $\bar{\mu}$ can be estimated as mean of the observed values, with the precision depending on sample size. But can we restore the noise summand σ_ε^2 from the estimate of standard deviation?

Probability mass functions of normal distributions are the same if and only if both parameters μ and σ are the same for both distributions. The only information about variance that can be drawn from a sample is linear sum of squares $\bar{\sigma}^2 + \sigma_\varepsilon^2$. Dimension of solutions of the equation $\bar{\sigma}_1^2 + \sigma_{\varepsilon 1}^2 = \bar{\sigma}_2^2 + \sigma_{\varepsilon 2}^2$ is 1, so we cannot uniquely determine σ_ε (**Suppl. Fig. SN 1.1**).

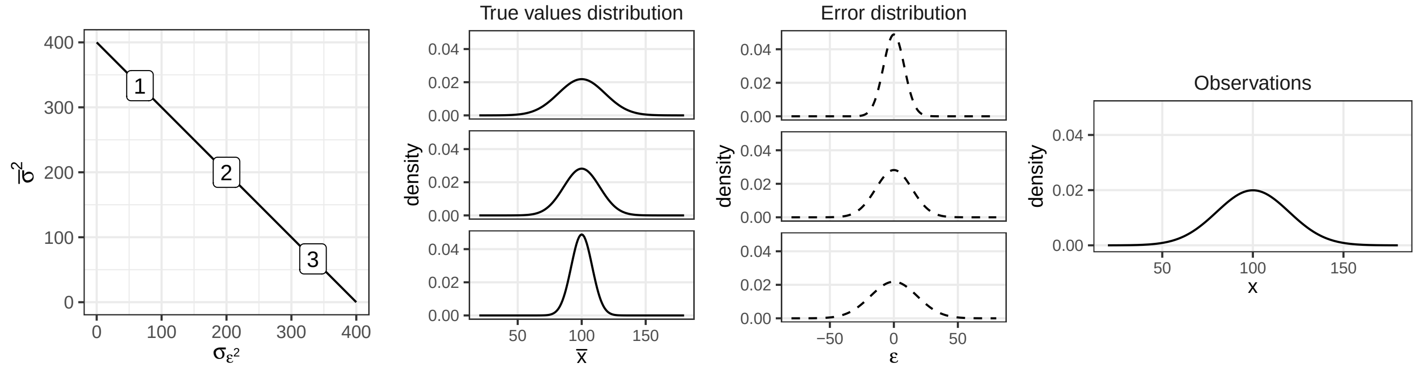

Supplementary Figure SN 1.1: Different parametrizations of signal and error distributions resulting in the same distribution of observations.

The situation substantially changes if second measurement is provided, and we can consider $\Delta x = x_1 - x_2$ distribution

$$x_1, x_2 \sim \mathcal{N}(\bar{x}, \sigma_\varepsilon^2)$$

$$x_1 - x_2 \sim \mathcal{N}(0, 2 \cdot \sigma_\varepsilon^2)$$

where parameter σ_ε becomes uniquely computable.

1.2. Signal: trimodal delta or beta distribution; noise: beta-binomial or binomial

One of the popular models for the allele-specific analysis is based on trimodal state assumptions on true AI values: biallelic or completely biased towards one of the alleles, and considers the variation coming from Beta-Binomial noise [3, 5].

For example, one may fit the observed distribution with mix of two symmetric Beta-Binomial distributions (**Suppl. Fig. SN 1.2**), considering values assigned to convex-shape Beta-Binomial distribution ($\alpha_1 = \beta_1 > 1$) coming from 50:50 and values assigned to concave-shape Beta-Binomial distribution ($1 > \alpha_2 = \beta_2 > 0$) coming from 100:0 or 0:100.

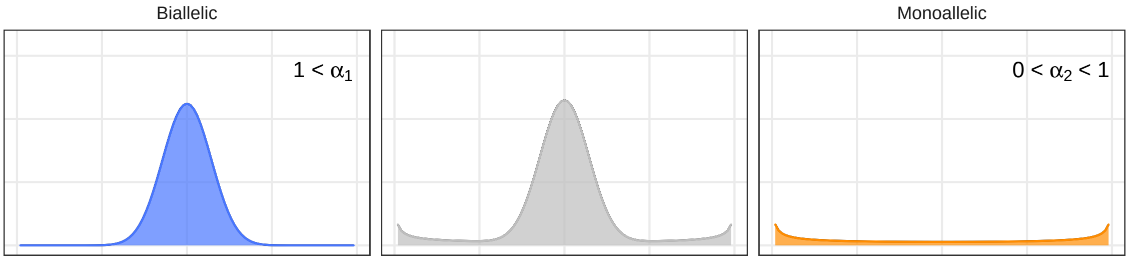

Supplementary Figure SN 1.2: Schematic representation of the distributing genes according to the fit of convex-shape and concave-shape mixture distribution.

Then this, by design, is indistinguishable from the case when we, oppositely, consider sampling without overdispersion (Binomial distribution), but true AI values coming from mixture of two Beta distributions with corresponding α_1 and α_2 (**Suppl. Fig. SN 1.3**).

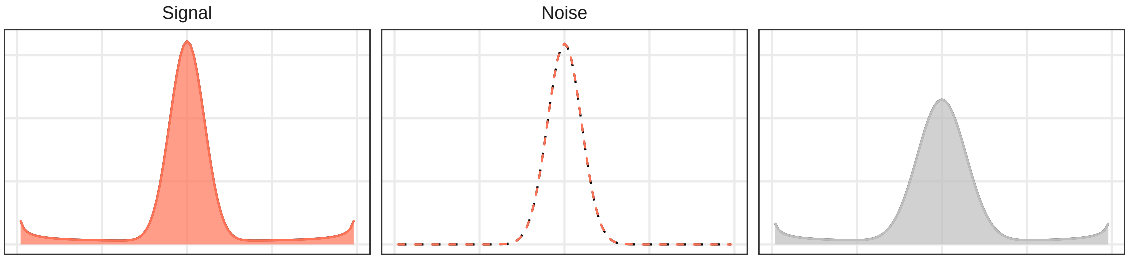

Supplementary Figure SN 1.3: Second parametrization that results in the same distribution of observations: beta distributed signal and binomial noise.

Note that if parameters α_1 and α_2 are fixed, then the distributions constructed above would converge to different states as $n \to \infty$, namely to the trimodal Dirac distribution and Beta distribution, and thus may potentially be distinguished on genes with different coverage.

However, we observe that extra-binomial variation remains constant for any coverage (see **Fig. 3 and Suppl. Fig. S14**). To reflect this, one may consider shape parameters being functions of n to make the limits similar to the first model, i.e. trimodal Delta function, for example (**Suppl. Fig. SN 1.4**):

$$\alpha_1(n) = \alpha_{01} \cdot \ln(n)$$

$$\alpha_2(n) = \frac{\alpha_{02}}{\ln(n)}$$

for some constants α_{01} and α_{02}.

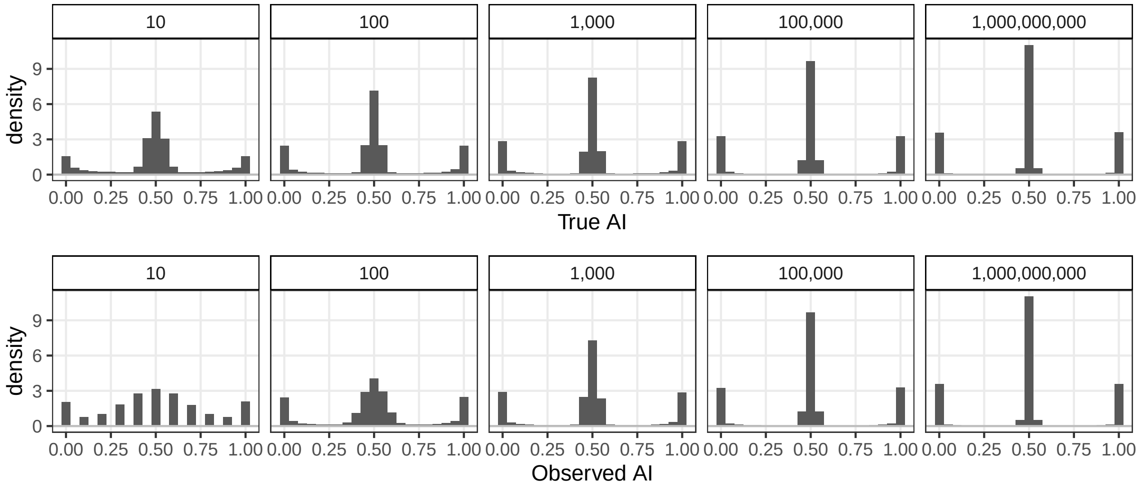

Supplementary Figure SN 1.4: An example of Beta mixture and respective Beta-Binomial mixture, which converge to trimodal distribution with coverage tends to infinity ($w_1 = 0.6$, $\alpha_1 = 20$, $\alpha_2 = 19/20$, correction: $\log_2(\text{cov})$)

With α_i depending on n these two Beta-Binomial distributions become indistinguishable.

1.3. Case of beta-binomial distributed signal and noise

Now, let both true AI values and noise belong to the Beta-Binomial distributions family. It is a common assumption that noise can be considered Beta-Binomial [1, 2, 3, 4], and this class provide reasonable bell curves that may be used to model true AI values distribution. Then, for some coverage C and a pair of overdispersion parameters ρ_1 and ρ_2:

$$\text{AI}_{\text{obs}} \sim \text{Beta-Bin}(C, p = \text{AI}_{\text{true}}, \rho_2)$$

where

$$\text{AI}_{\text{true}} \sim \text{Beta-Bin}(C, p = 0.5, \rho_1)$$

The relation between signal and noise is not symmetric, and the "complementary" distributions are not equal:

$$\text{Beta-Bin}(C, \text{Beta-Bin}(C, 0.5, \rho_1), \rho_2) \neq \text{Beta-Bin}(C, \text{Beta-Bin}(C, 0.5, \rho_2), \rho_1)$$

for $\rho_1 \neq \rho_2$ (**Suppl. Fig. SN 1.5**). We will use these pairs of "complementary" distributions to illustrate the idea that completely different parametrizations (that cannot be considered uniformly close) may result in similar observations.

	0	1	2	3	4	5	6	7	8	9	10	11	12	13	14	15
**BB2(BB1)**	0.015	0.029	0.045	0.06	0.074	0.086	0.094	0.098	0.098	0.094	0.086	0.074	0.06	0.045	0.029	0.015
**BB1(BB2)**	0.017	0.028	0.043	0.059	0.074	0.086	0.095	0.099	0.099	0.095	0.086	0.074	0.059	0.043	0.028	0.017

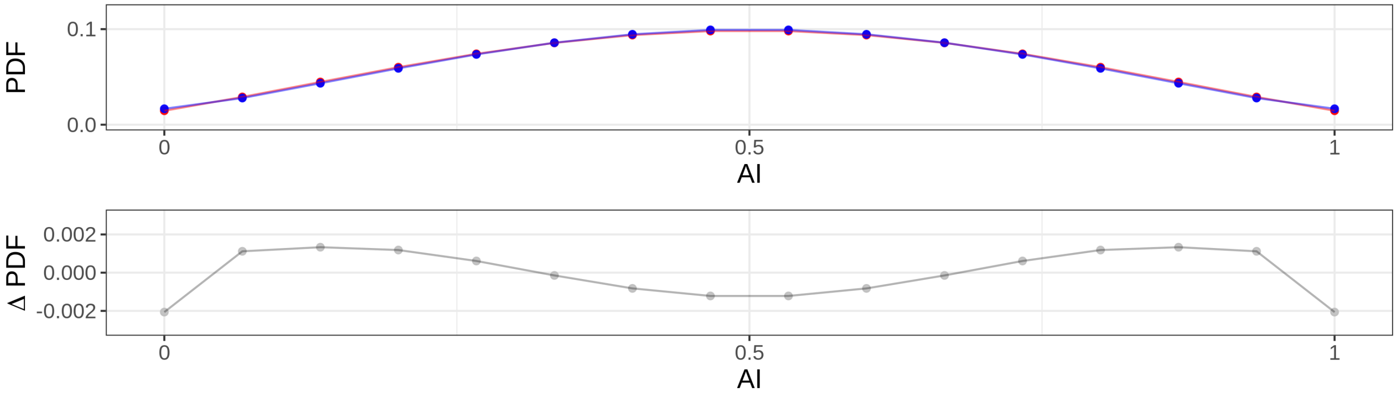

Supplementary Figure SN 1.5: An example of non-equality of two "complementary" distributions (*red* and *blue*). While probability density function (PDF) values appear similar (*top plot*), they are not the same (*table* and *bottom plot*). Distributions plotted: Beta-Bin(C, Beta-Bin(C, 0.5, ρ_1), ρ_2) (denoted *BB2(BB1)*) and Beta-Bin(C, Beta-Bin(C, 0.5, ρ_2), ρ_1) (denoted *BB1(BB2)*): $n = 15$, $\rho_1 = 0.01$, $\rho_2 = 0.1$.

Importantly, in general the samples from "complementary" distributions are indistinguishable (Probability Density Functions and QQ-plots for "complementary" distributions: **Suppl. Fig. SN 1.6**), and the distributions of p-values of Mann-Whitney-Wilcoxon test are indistinguishable from uniform distributions (**Suppl. Fig. SN 1.7a**), when distributions of p-values of Kolmogorov-Smirnov test are similar for distributions with the same and "complementary" parametrization (**Suppl. Fig. SN 1.7b**).

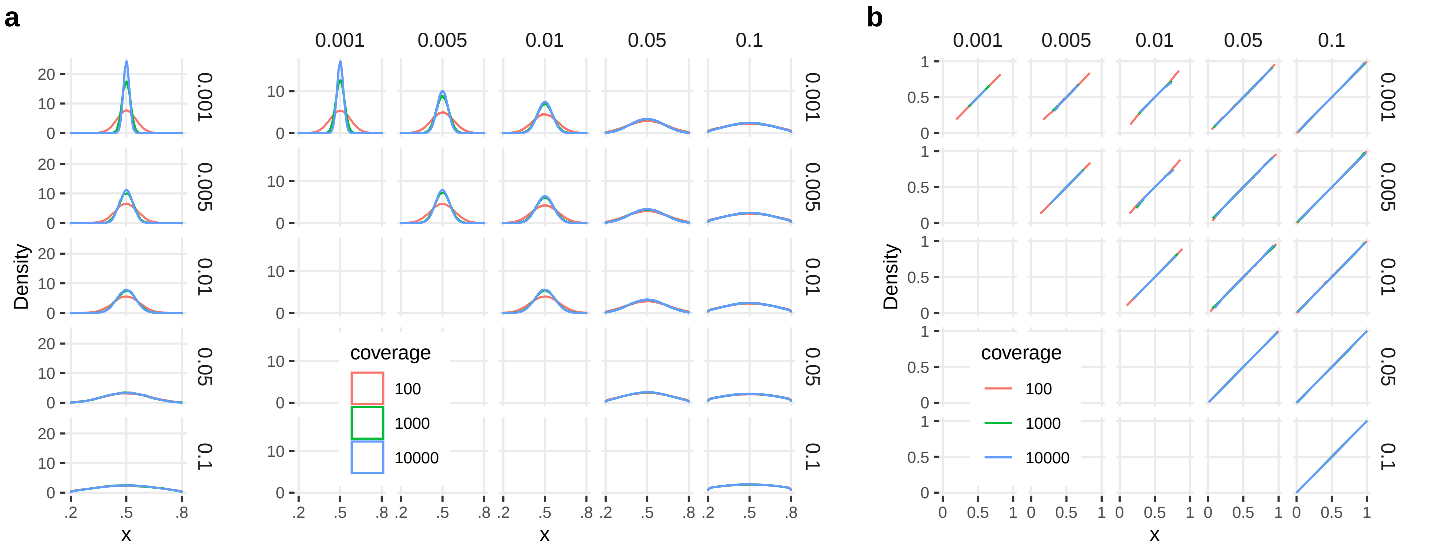

Supplementary Figure SN 1.6: Examples of "complementary" distributions ($\rho_{11} = \rho_{22}$ and $\rho_{21} = \rho_{12}$) for different pairs of ρ_1, ρ_2 and coverage levels. **(a)** Density plots, **(b)** QQ-plots between complementary distributions.

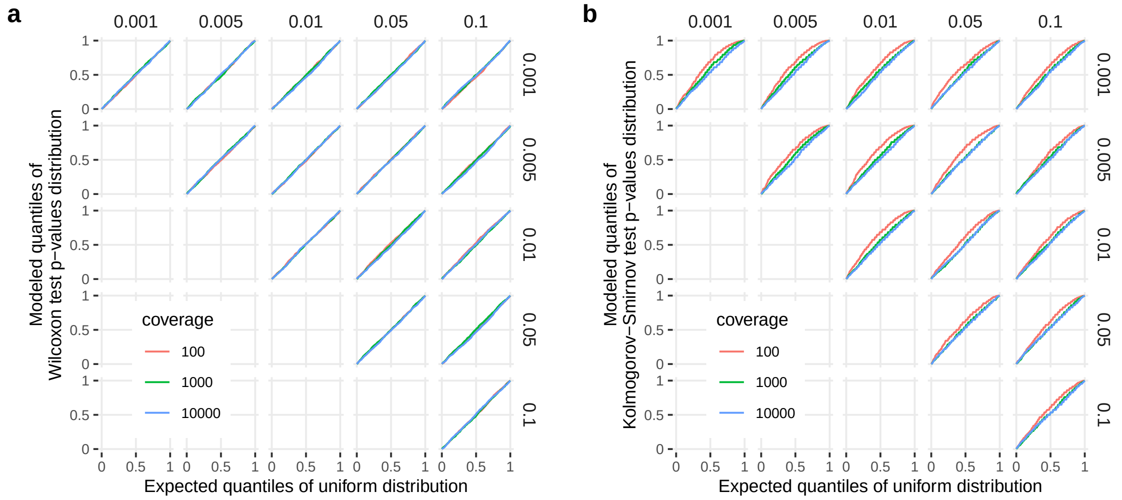

Supplementary Figure SN 1.7: QQ-plots for uniform distribution and the distributions of **(a)** Two-sided Mann-Whitney-Wilcoxon test p-value statistics and **(b)** Two-sample Kolmogorov-Smirnov test p-value statistics on the "complementary" distributions ($\rho_{11} = \rho_{22}$ and $\rho_{21} = \rho_{12}$).

1.4. General critique of using fixed AI distribution

All the approaches to AI analysis of RNA-seq start with the assumption that a specific distribution from a preset distribution family is a good general approximation for the whole experiment. However, a simple thought experiment shows that AI distribution can stop belonging to a particular distribution family; more generally, that it may not be appropriate to assume that any particular distribution is a universal fit to all genes in an experiment.

To illustrate the first point, consider a perturbation (e.g., drug treatment) that changes allelic imbalance in a large fraction of genes. After the treatment the genes would be divided into those belonging to the "old" distribution, and the affected genes that belong to the new one. As an example of such perturbation, let half the genes respond by a decrease in AI: their AI values become half as far from 50:50 balance. If the initial distribution in this example is the beta distribution, the resulting mixture does not belong to beta family.

These considerations suggest that any particular distribution cannot be a universal fit to all experiments. Thus, instead of using a generalization of fitted AI distribution along the coverage, we considered separately each bin, aiming to reflect local states. We still needed a prior distribution family to use in order to mimic the replicates without overdispersion, so Beta distribution was used in each bin to capture the local AI estimates behaviour in each bin.

Supplementary Note S 2. Accounting for overdispersion leads to the expected bimodal distribution of AI values in discordant AI calls.

With what probability the binomial-like test (**Suppl. Fig. SN 2.1a**) would show different results on two maternal counts observations M_1 and M_2 for a given underlying proportion a (**Suppl. Fig. SN 2.1b**)?

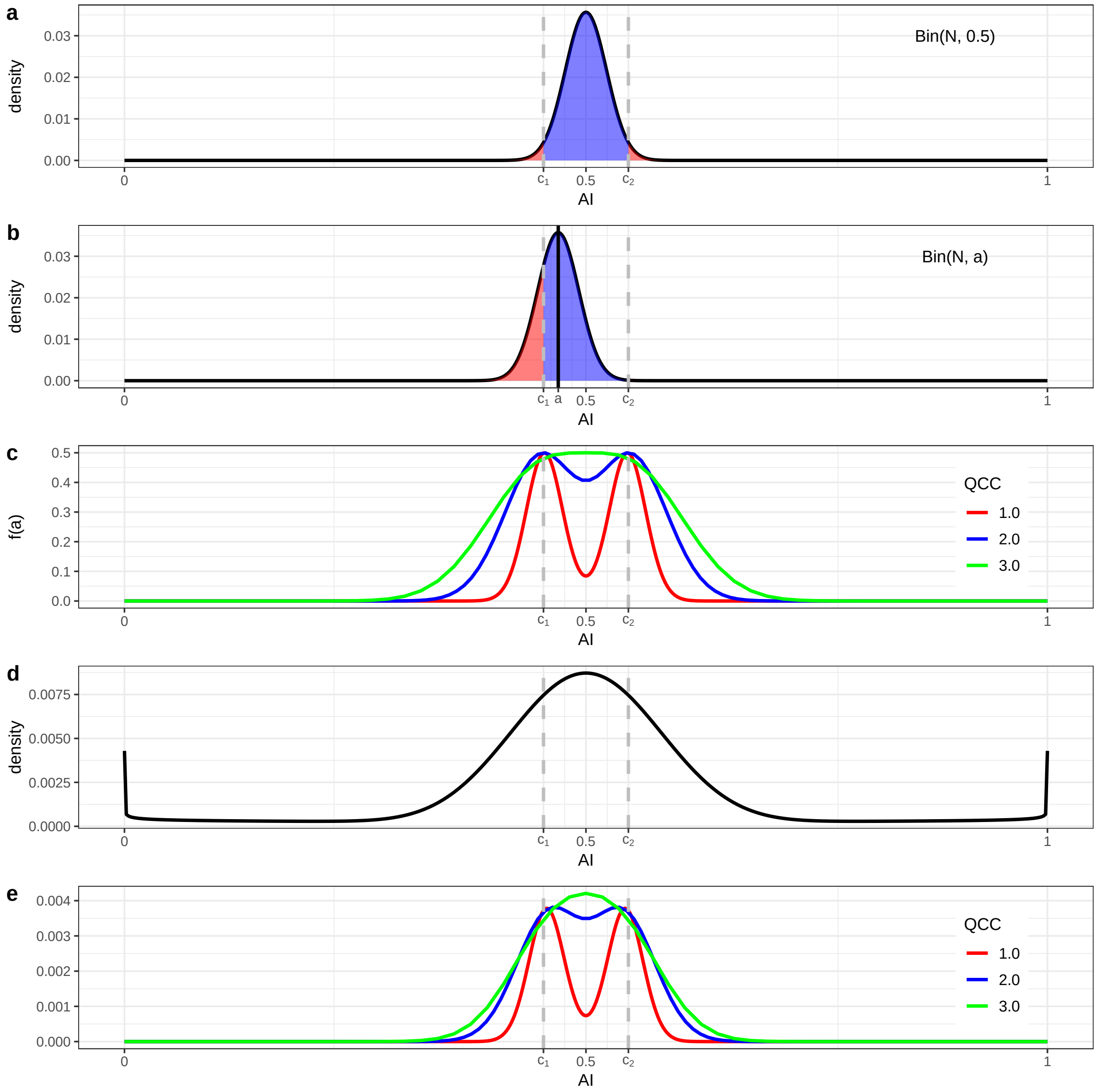

Supplementary Figure SN 2.1: (a) Binomial distribution and boundaries of binomial test with 0.5, for given $N = 500$ and level of confidence 0.95; **(b)** Binomial distribution of observed AI for underlying imbalance $a = 0.47$, colored according to binomial test result; **(c)** Probability to receive discordant test results for 2 observations for different QCC (recall that $QCC = 1$ corresponds to binomial distribution and thus represents the case when the test fits the data); **(d)** Sample distribution of underlying AI; **(e)** Distribution of underlying AI of genes that may be marked differently when doing binomial test for 2 technical replicates (for $QCC = 1$ we see that it is bimodal and the peaks are near binomial test boundaries).

Let us consider the function of that probability for particular gene coverage N and respective boundaries C_1 and C_2 of the binomial test BT_{QCC} with $H_0 : p = 0.5$, on counts respectivelly corrected on QCC (**Suppl. Fig. SN 2.1c**). Then probability of discordant True/False results of BT_{QCC} on 2 technical replicates for underlying proportions $a \in (0, 1)$ is:

$$f_{N,QCC}(a) = P(\text{BT}_{QCC}(M_1) \neq \text{BT}_{QCC}(M_2)) =$$

$$= 2 \cdot (P(M \leq C_1|a) \cdot P(M \in (C_1, C_2)|a) + P(M \geq C_2|a) \cdot P(M \in (C_1, C_2)|a)) =$$

$$= 2 \cdot P(M \in (C_1, C_2)|a) \cdot (P(M \leq C_1|a) + P(M \geq C_2|a)) =$$

$$= 2 \cdot \int_{C_1}^{C_2} \text{Bin}_{QCC}(x; N, a)dx \cdot (\int_{0}^{C_1} \text{Bin}_{QCC}(x; N, a)dx + \int_{C_2}^{N} \text{Bin}_{QCC}(x; N, a)dx)$$

Given discretized distribution $U_N(a)$ of underlying AI (**Suppl. Fig. SN 2.1d**), we may obtain the distribution of AI of genes with discordant results of BT_{QCC} on 2 technical replicates, as a product of $U_N(a)$ and $f_{N,QCC}(a)$ (**Suppl. Fig. SN 2.1e**).

Note that if the test uses the distribution which fits the data well, the AI values of genes with discordant results will be distributed around test boundaries (**Suppl. Fig. SN 2.3**). By contrast, not accounting for overdispersion tends to much wider, sometimes even unimodal distribution, which is what we observe on our data (**Suppl. Fig. SN 2.2**, see also **Fig.2b,f** in the main text).

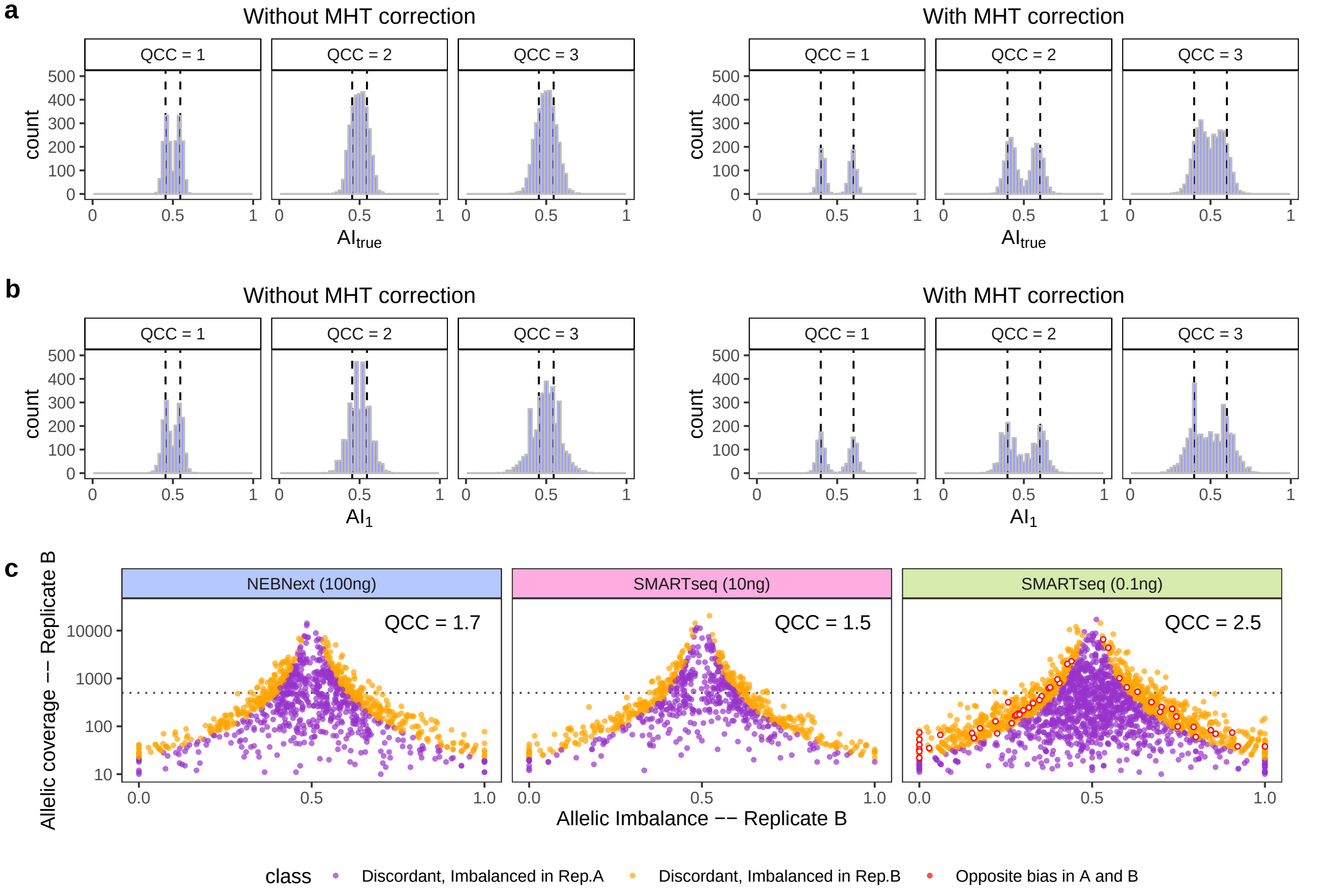

Supplementary Figure SN 2.2: Testing for null hypothesis of allelic imbalance with no correction for overdispersion. **(a)(b)** Simulated data. Distributions of **(a)** true AI values AI_{true} and **(b)** observed AI values from one replicate (AI_1) for genes with discordant imbalance calls in two simulated replicates. Shown are simulated data with 10,000 genes with coverage 500 and true AI values distributed as 85:15 mixture of beta distributions with $\alpha_1 = \beta_1 = 20$ and $\alpha_1 = \beta_1 = 0.8$; 95% confidence; whether MHT correction (Bonferroni) was applied is noted). Note that the differences in the shapes of the AI distributions reflect the difference in data overdispersion level, as described above (**Suppl. Fig. SN 2.1e**). **(c)** AI discordance maps for experimental data (same data as in **Fig.2b** in the main text). Panels **a** and **b** (with MHT correction) represent density plots along the dotted line at coverage of 500.

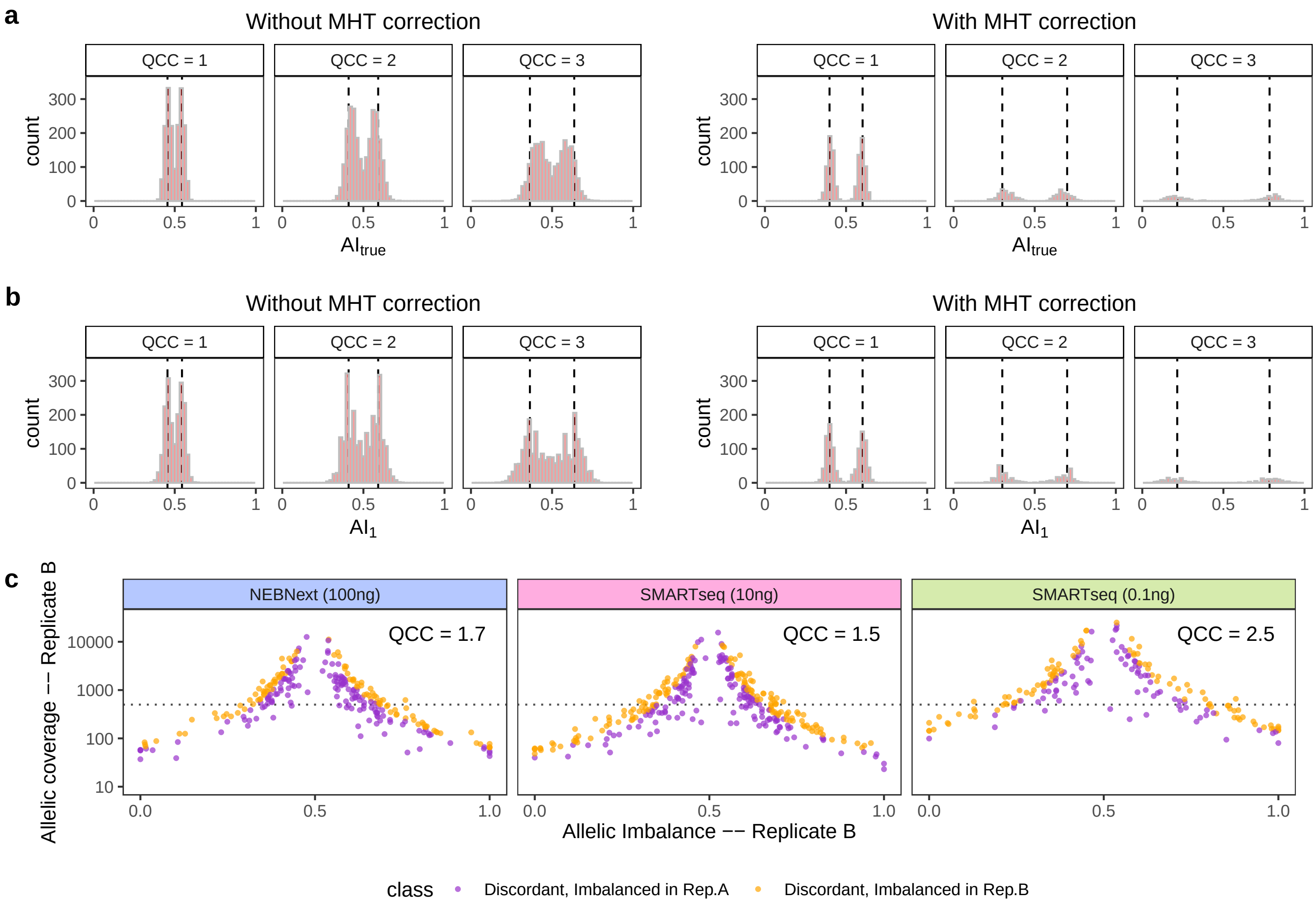

Supplementary Figure SN 2.3: Same analysis as in **Suppl. Fig. SN 2.2**, but with AI overdispersion accounted for. Note that both distributions of AI_{true} **(a)** and AI_1 **(b)** are much clearer distributed around test boundaries. The same is observed on the replicate data **(c)** when QCC correction is applied (see also **Fig.2** in the main text).

Supplementary Note S 3. Genes with different underlying AI have different impact on the overall signal variance.

Let us consider genes in particular coverage bin.

If AI values for 2 given replicates, $x_1 = \{x_{1i}\}$ and $x_2 = \{x_{2i}\}$, belong to the same binomial-like distribution and corresponding underlying allelic proportions $a = \{a_i\}$ belong to any symmetric distribution, then since $var(x_{1i} - x_{2i}) = var(x_{1i}) + var(x_{2i})$ we expect that $var(x_{1i} - x_{2i}) \sim a_i(1 - a_i)$ for any gene i.

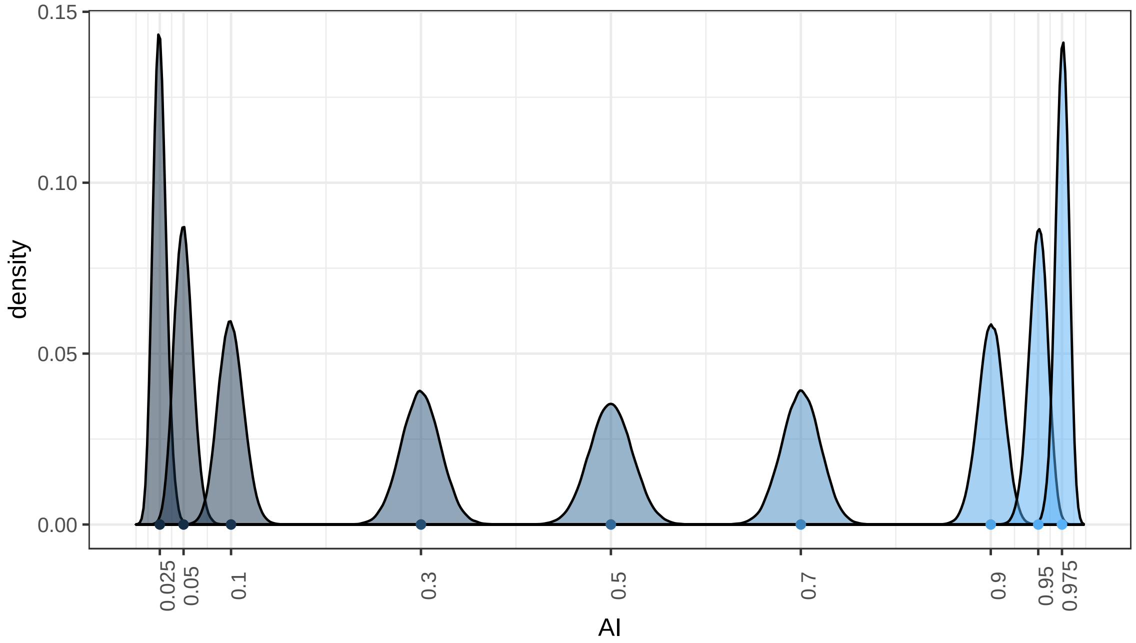

Supplementary Figure SN 3.1: Variance of AI observations differs along the interlval of underlying AI (gene coverage 500).

And $a_i(1 - a_i)$ reaches its maximum at 0.5 and minimum at 0 and 1 (**Suppl. Fig. SN 3.1**).

For $X = \{x_{1i} - x_{2i}\}_i$ then $var(X) = E((X - \mu)^2) = E((X - 0)^2) = \frac{1}{n}\sum_{i=1}^{n}(x_{1i} - x_{2i})^2$, which depend on the distribution of underlying allelic proportions $a = \{a_i\}$.

And since underlying AI belong to some complex distribution, and genes with different underlying AI have different impact on the overall signal variance, the AI distribution should be taken into account when making any conclusions about distribution of differences between AI observations in technical replicates.

Thus we conclude that it is necessary to account for the distribution of underlying allelic imbalances when assessing AI differences between technical replicates, while assumption of a simple distribution (e.g. tri-modal) is insufficient for this purpose. We account for the observed distribution in the beta-binomial model (see **Fig. 3d**).

Supplementary Note S 4. We expect nearly zero genes with false positive AI, when we estimate AI and CI from two replicates and then calculate AI from six replicates.

Here, we define "false positive" as the event when the point estimate of AI for a gene from six replicates is outside of the CI for the same gene based on two replicates out of six (after Bonferroni correction).

For particular gene and respective underlying AI p, maternal counts in n replicates are expected to follow the same distribution $m_{i\in\{1..n\}} \sim \mathrm{Bin}(C,p)$ (we consider here the case of QCC $=1$, but it can be clearly generalized). Then for CI($\mathrm{AI}_2 = \frac{m_1+m_2}{2\cdot C}$) computed on the first 2 replicates, the probability of underlying AI p to miss CI(AI_2) (which is determined by confidence level by design, for example, 0.05) is less than probability of AI estimate on k replicates that are different from the first 2 replicates ($\frac{\sum_3^{k+2} m_i}{k\cdot C} \sim \frac{\mathrm{Bin}(k\cdot C,p)}{k\cdot C}$), but is greater than probability of CI(AI_2) to be missed by AI estimate computed on k replicates that include the first 2 replicates ($\frac{\sum_1^{k} m_i}{k\cdot C} \sim \frac{\mathrm{Bin}(k\cdot C,p)}{k\cdot C}$), and not independent with $\frac{m_1+m_2}{2\cdot C}$).

In our case:

$$P\left(\frac{\sum_1^6 m_i}{6\cdot C} \notin \mathrm{CI}\left(\frac{m_1+m_2}{2\cdot C}\right)\right) \leq P\left(p \notin \mathrm{CI}\left(\frac{m_1+m_2}{2\cdot C}\right)\right) \leq P\left(\frac{\sum_3^8 m_i}{6\cdot C} \notin \mathrm{CI}\left(\frac{m_1+m_2}{2\cdot C}\right)\right)$$

Simulations supporting this idea are provided in **Suppl. Fig. SN 4.1** and **Suppl. Fig. SN 4.2**.

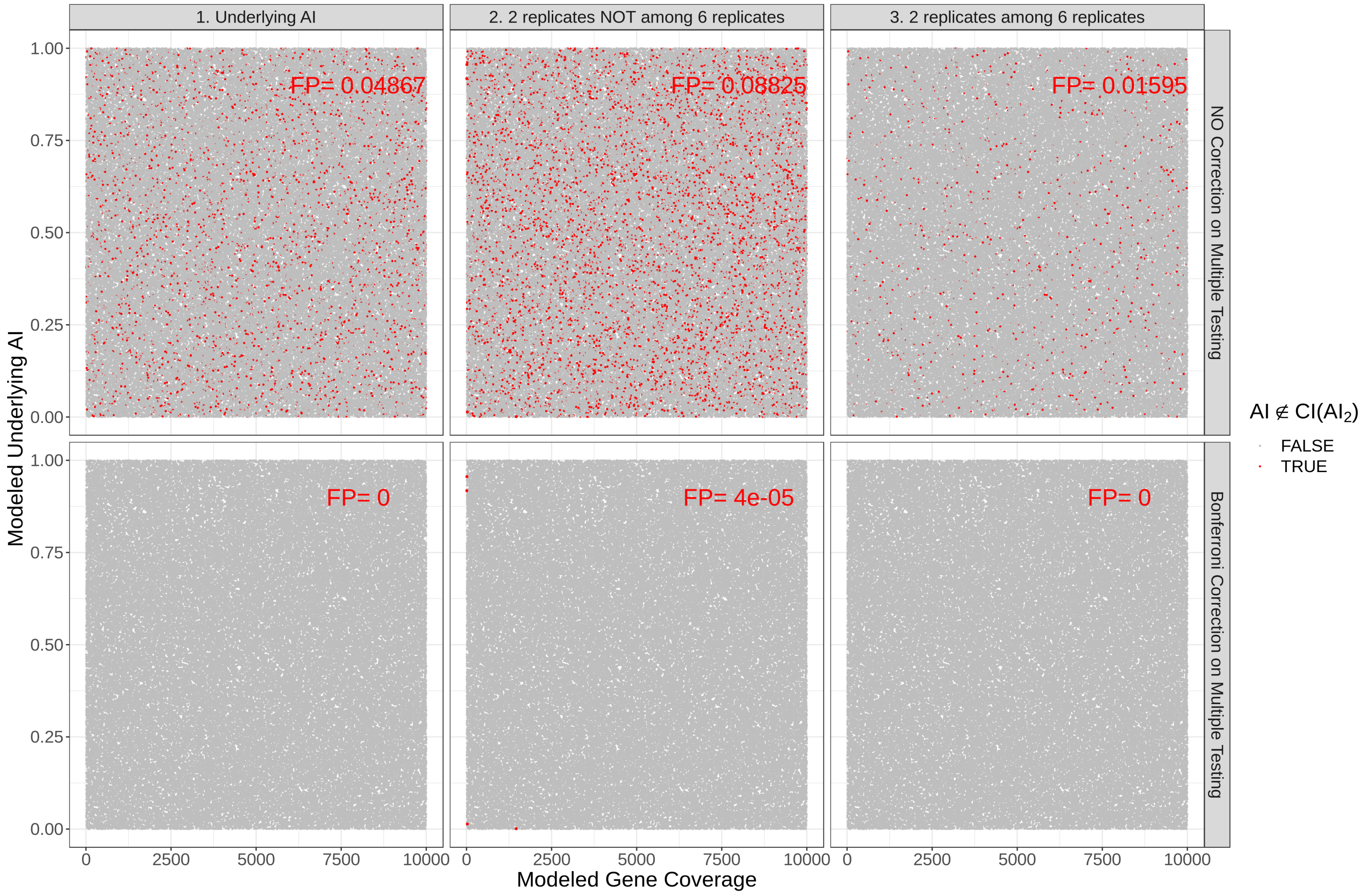

Supplementary Figure SN 4.1: Number of false positives for modeled 100,000 genes with different coverage and underlying AI. The point used to test if it lies within $CI(AI_2)$ calculated on 2 randomly selected replicates, left to right: underlying AI, AI estimate from a set of 6 replicates that are different from initial 2 replicates, AI estimate from a set of 6 replicates which contain initial 2 replicates. The false positive rate is provided before and after correction on multiple testing.

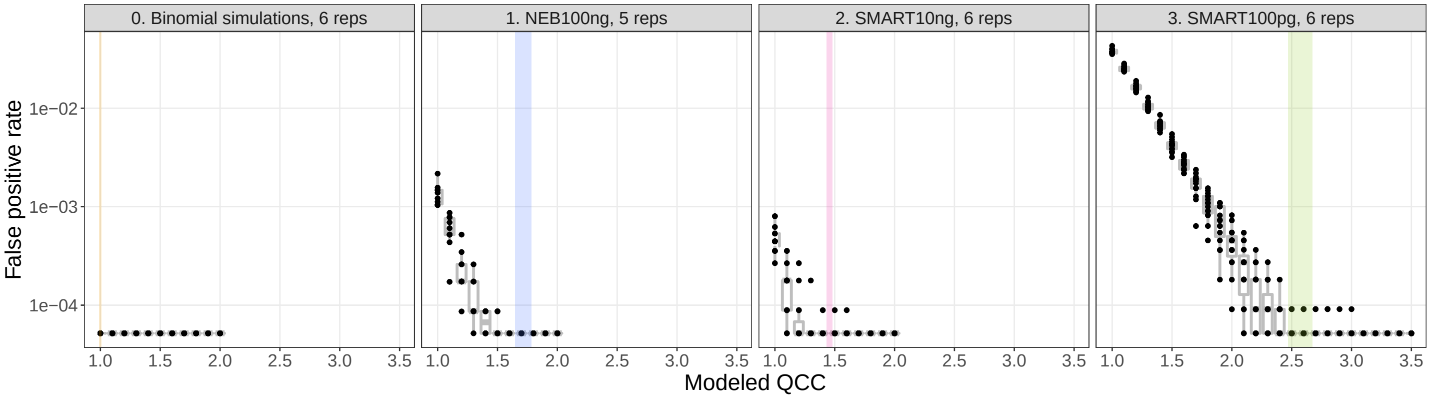

Supplementary Figure SN 4.2: False positive rate for binomial simulations ($QCC = 1$) and different experiments for different modeled QCC. The range of real QCC is colored.

Supplementary Note S 5. Statistical power of the QCC-corrected test.

We showed that QCC correction dramatically lowers false positive (FP) rate. A natural question is what is the trade-off in terms of the loss of signal. Note that "true positive" is more challenging to determine than FP. An intuition can be given by pixels forming an image: with unlimited resolution, any two distinct dots can be resolved. However, any specific resolution imposes a limit on the ability to resolve dots. Library complexity is equivalent to resolution while coverage depth is equivalent to magnification. In other words, whether two AI values are distinguishable is a property of the data, and power analysis is an estimation of necessary coverage for a particular needed level of resolution with a given quality of the data.

5.1. As a measure of "signal", we will use statistical power, a common measure used to estimate the required sample size that allows detection of the true difference at the scale of interest with a given significance [6]. Let us consider a two-sample differential AI problem: if we have a gene such that ΔAI is some specific value between samples 1 and 2, and AI_0 is the mean of these values, what is the chance we will not detect it as having significant difference in AI, given this gene's coverage and technical overdispersion (same for both samples), with desired level of confidence (**Suppl. Fig. SN 5.1**).

Note that comparison of a single sample AI to a specific AI value is a formally equivalent problem, but the two-sample problem has much more clear interpretation in case of biologically relevant continuous distribution of AI values in samples.

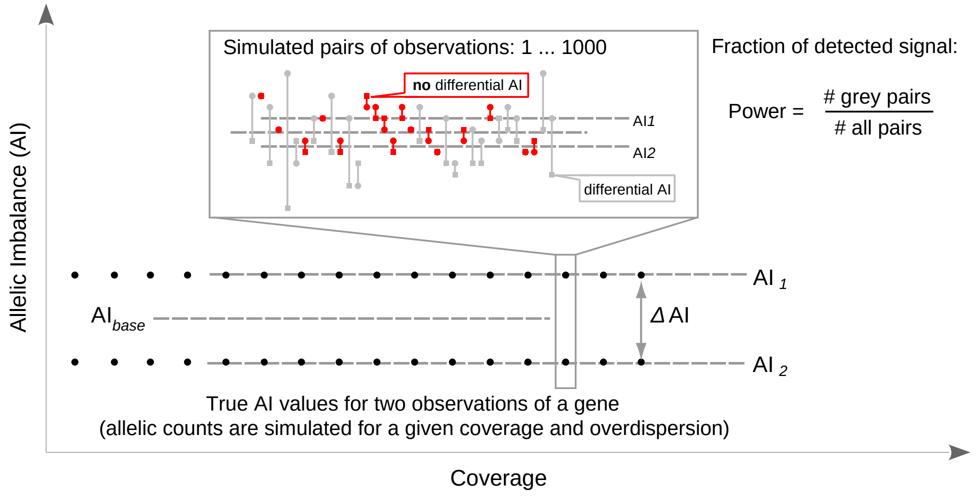

Supplementary Figure SN 5.1: Schematic definition of statistical power (i.e., fraction of detected signal for particular level of resolution).

Statistical power depends on allelic coverage and 4 additional parameters, defining specific H_1 hypothesis:

- base AI (AI_0) and AI difference (ΔAI), which will determine AI_1 and AI_2 as $\mathrm{AI}_0 \pm \frac{\Delta \mathrm{AI}}{2}$, used in data simulation;
- overdispersion parameter $\mathcal{O}$ of the data, used in data simulation;
- QCC used in the test (Binomial $\Leftrightarrow$ QCC $= 1$; QCC-corrected test $\Leftrightarrow$ QCC $= \sqrt{\mathcal{O}}$)

To approximate power values $\mathcal{P}(\mathrm{cov}_i \mid \mathcal{O}, \mathrm{QCC}, \mathrm{AI}_0, \Delta\mathrm{AI})$ in this analysis, we generate 1000 AI pairs $\{\mathrm{AI}_{ijk}\}_{k \in 1\ldots1000}$ from $\mathrm{Bin}(\frac{\mathrm{cov}_i}{\mathcal{O}}, \mathrm{AI}_j)$ (for $j \in \{1, 2\}$) distribution for different coverage levels cov_i, and take the percentage of events when the differential test returned a positive result for AI_{i1k} and AI_{i2k} (differential at the selected confidence level, here: 0.95).

Note once again that statistical power is not a comprehensive measure of true positive calls, since "resolution" of this measure depends on fixed AI_0 and ΔAI, and thus may be used only for estimation of necessary coverage for

a particular needed level of resolution.

5.2. A measure of FP (false positives) is defined in the regular way, in a complementary manner to statistical power: how many of the identical points are classified as having significant difference in AI. To illustrate the effect of underestimation of overdispersion on signal detection, we will compare our method with binomial test in this note. **Suppl. Fig. SN 5.2** shows that FP rate remains nearly constant across coverage, and overdispersion leads to increased FP rate when using binomial test (i.e. insufficient correction when assuming no technical overdispersion). QCC-corrected test controls for this extra-binomial variance.

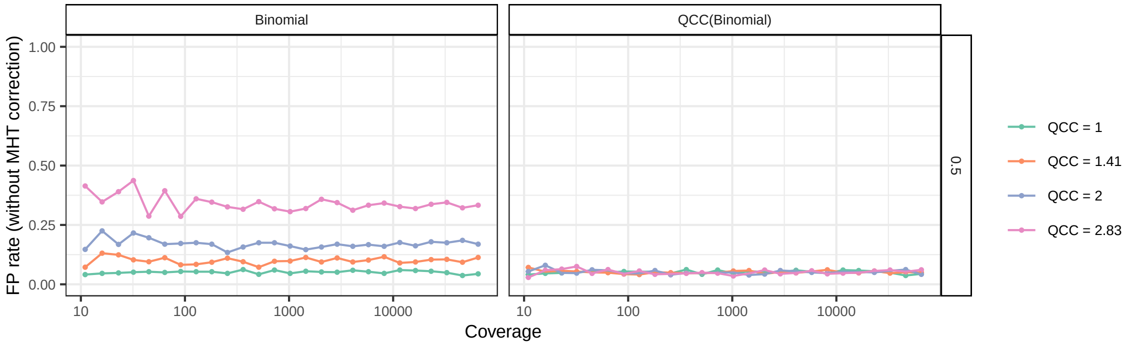

Supplementary Figure SN 5.2: False positive rate of differential tests with binomial and QCC-corrected assumptions, shown for the data generated with different overdispersion (marked with color), for different coverage levels and the particular allelic proportion AI = 0.5.

5.3. Having constructed these measures, we can explore their dependence on coverage and overdispersion (**Suppl. Fig. SN 5.3**).

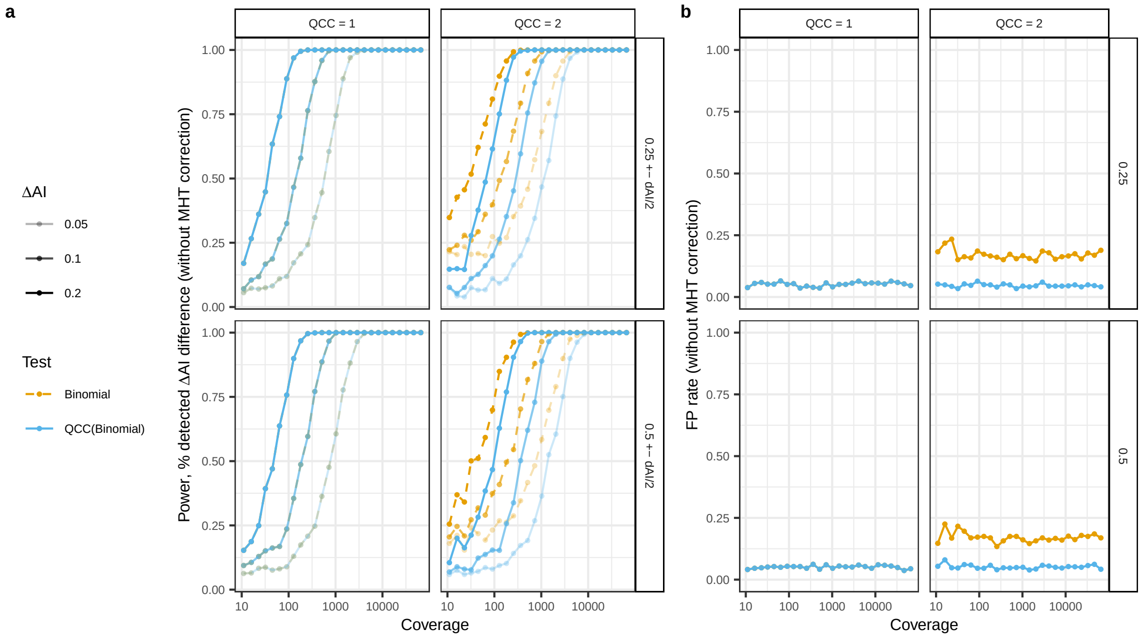

Supplementary Figure SN 5.3: Statistical power **(a)** and false positive rate **(b)** of differential tests with binomial and QCC-corrected assumptions, shown for the data generated with different overdispersion (columns, QCC = 1 and QCC = 2), for different coverage levels and two example allelic proportions (rows, AI = 0.25 and AI = 0.5). When QCC = 1, differential tests values coincide. **(a)** Level of transparency reflects difference ΔAI in true AI values between two generated samples.

With no technical overdispersion, binomial test and QCC-corrected test coincide on the same dataset. When overdispersion is present in the data, at low coverage, the test which takes underestimated overdispersion shows the higher power than QCC-corrected test (**Suppl. Fig. SN 5.3.a**), which goes together with the unavoidable cost

of the increased FP rate (**Suppl. Fig. SN 5.3.b**). Note that power of differential test with binomial assumptions doesn't get close to 0 at the low coverage (**Suppl. Fig. SN 5.3.a**). The difference in power values between binomial and QCC-corrected tests diminishes with increase in coverage, and for both tests statistical power reaches 1.0 for genes with coverage over some level. Obviously, larger ΔAI is resolved better at any given coverage level, and sufficiently small ΔAI cannot be resolved at low coverage level (**Suppl. Fig. SN 5.4**).

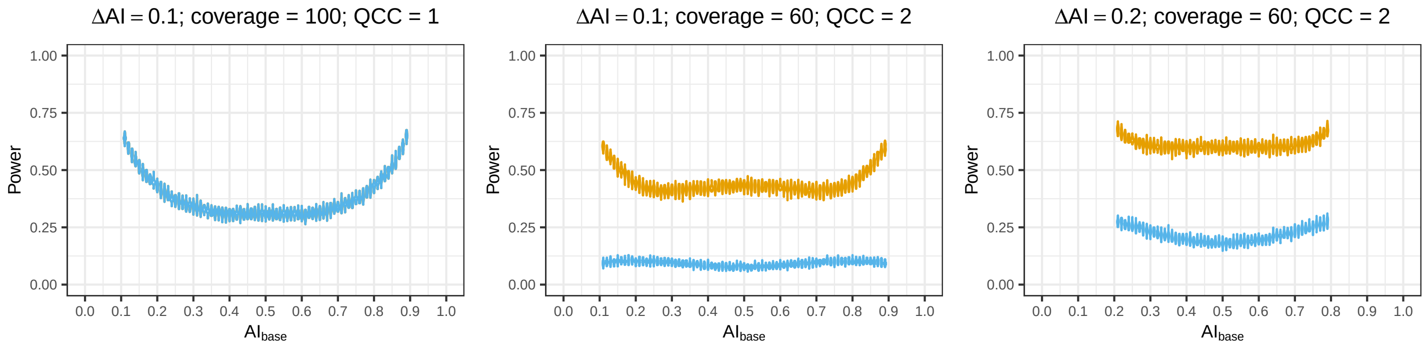

Supplementary Figure SN 5.4: Statistical power of differential tests with binomial (orange) and QCC-corrected assumptions (blue), shown for the data generated with several sets of parameters: overdispersion (QCC), coverage level and difference in true AI values (ΔAI). Each violin plot corresponds to comparison between samples with true AI values $\mathrm{AI}_{\mathrm{base}} - \frac{\Delta \mathrm{AI}}{2}$ and $\mathrm{AI}_{\mathrm{base}} + \frac{\Delta \mathrm{AI}}{2}$. When QCC = 1, differential tests values coincide.

5.4. An important practical conclusion is that QCC-corrected analysis greatly reduces FPs, and misses little true signal for all genes with coverage over some threshold. The "cost" in power decreases with coverage and eventually disappears (**Suppl. Fig. SN 5.5**). Critically, coverage can be easily increased by additional sequencing. By contrast, for binomial test FP cost remains constant non-zero, regardless of coverage.

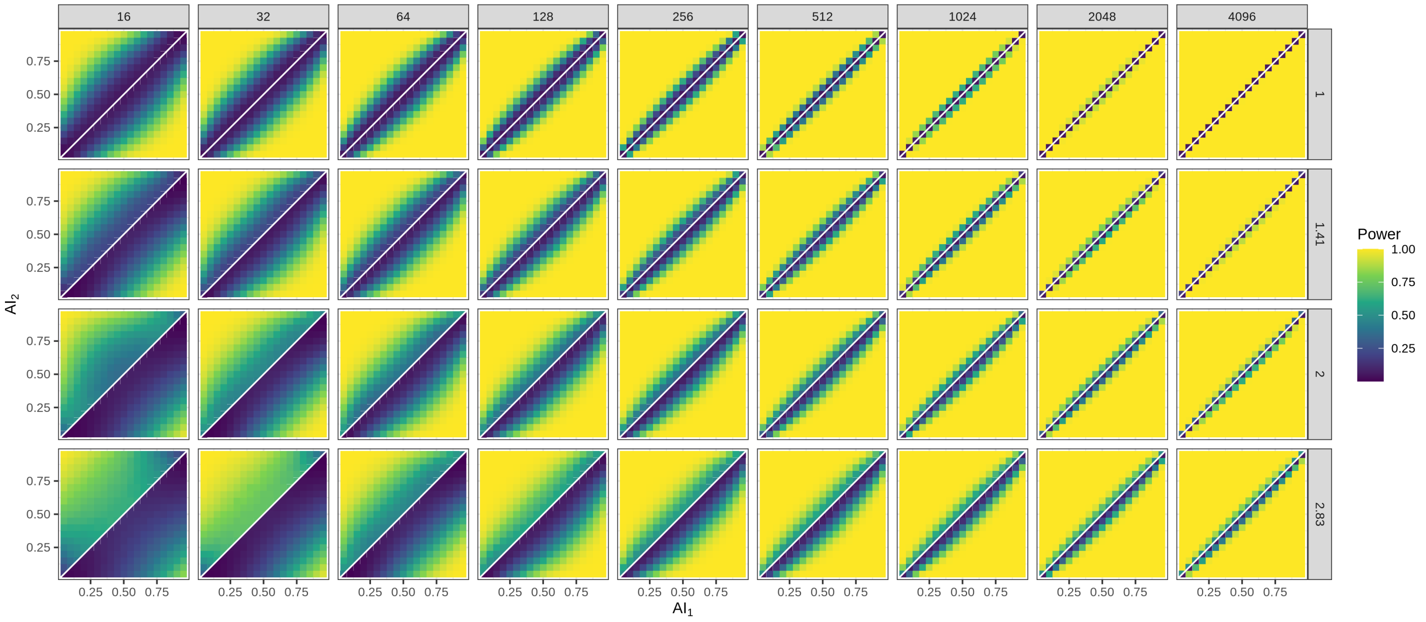

Supplementary Figure SN 5.5: Heatmaps of statistical power of differential tests with binomial (upper-left triangle at each plot) and QCC-corrected assumptions (bottom-right triangle), shown for the data generated with several sets of parameters: overdispersion (rows, QCC), coverage level (columns) and true AI values (axis, AI_1 and AI_2) used in generation samples for comparison.

Supplementary Note S 6. Worked example of QCC calculation, starting from fastq.

fastq → counts:
https://github.com/gimelbrantlab/ASEReadCounter_star/wiki/2.-Allelic-Counts-Table-Creation

counts → QCC:
https://github.com/gimelbrantlab/Qllelic/wiki/Use-case-1:-One-biological-sample

Supplementary Note S 7. Worked example of AI differential analysis for two samples.

https://github.com/gimelbrantlab/Qllelic/wiki/Use-case-2:-Differential-AI-analysis

Supplementary References

[1] Skelly DA, Johansson M, Madeoy J, Wakefield J, Akey JM. *A powerful and flexible statistical framework for testing hypotheses of allele-specific gene expression from RNA-seq data.* Genome Res. 2011 Oct;21(10):1728-37. doi: 10.1101/gr.119784.110. Epub 2011 Aug 26. PMID: 21873452; PMCID: PMC3202289.

[2] Mayba O, Gilbert HN, Liu J, Haverty PM, Jhunjhunwala S, Jiang Z, Watanabe C, Zhang Z. *MBASED: allele-specific expression detection in cancer tissues and cell lines.* Genome Biol. 2014 Aug 7;15(8):405. doi: 10.1186/s13059-014-0405-3. PMID: 25315065; PMCID: PMC4165366.

[3] Harvey CT, Moyerbrailean GA, Davis GO, Wen X, Luca F, Pique-Regi R. *QuASAR: quantitative allele-specific analysis of reads. Bioinformatics.* 2015 Apr 15;31(8):1235-42. doi: 10.1093/bioinformatics/btu802. Epub 2014 Dec 4. PMID: 25480375; PMCID: PMC4393517.

[4] Edsgärd D, Iglesias MJ, Reilly SJ, Hamsten A, Tornvall P, Odeberg J, Emanuelsson O. *GeneiASE: Detection of condition-dependent and static allele-specific expression from RNA-seq data without haplotype information.* Sci Rep. 2016 Feb 18;6:21134. doi: 10.1038/srep21134. PMID: 26887787; PMCID: PMC4758070.

[5] van de Geijn B, McVicker G, Gilad Y, Pritchard JK. *WASP: allele-specific software for robust molecular quantitative trait locus discovery.* Nat Methods. 2015 Nov;12(11):1061-3. doi: 10.1038/nmeth.3582. Epub 2015 Sep 14. PMID: 26366987; PMCID: PMC4626402.

[6] Reinhart A. *Statistics Done Wrong: The Woefully Complete Guide.* No Starch Press. 2015
