# Supplementary material for: Replicate sequencing libraries are important for quantification of allelic imbalance
Source: Nat Commun. 2021 Jun 7;12:3370. doi: 10.1038/s41467-021-23544-8 (PMC8184992; doi:10.1038/s41467-021-23544-8)
Supplement: Supplementary file 3 — Reporting Summary [file 41467_2021_23544_MOESM3_ESM.pdf]

nature research

Corresponding author(s): Alexander Gimelbrant, Asia Mendelevich

Last updated by author(s): Apr 12, 2021

Reporting Summary

Nature Research wishes to improve the reproducibility of the work that we publish. This form provides structure for consistency and transparency in reporting. For further information on Nature Research policies, see our Editorial Policies and the Editorial Policy Checklist.

Statistics

For all statistical analyses, confirm that the following items are present in the figure legend, table legend, main text, or Methods section.

n/a	Confirmed	
☐	☒	The exact sample size (n) for each experimental group/condition, given as a discrete number and unit of measurement
☐	☒	A statement on whether measurements were taken from distinct samples or whether the same sample was measured repeatedly
☐	☒	The statistical test(s) used AND whether they are one- or two-sided<br>*Only common tests should be described solely by name; describe more complex techniques in the Methods section.*
☐	☒	A description of all covariates tested
☐	☒	A description of any assumptions or corrections, such as tests of normality and adjustment for multiple comparisons
☐	☒	A full description of the statistical parameters including central tendency (e.g. means) or other basic estimates (e.g. regression coefficient) AND variation (e.g. standard deviation) or associated estimates of uncertainty (e.g. confidence intervals)
☐	☒	For null hypothesis testing, the test statistic (e.g. F, t, r) with confidence intervals, effect sizes, degrees of freedom and P value noted<br>*Give P values as exact values whenever suitable.*
☒	☐	For Bayesian analysis, information on the choice of priors and Markov chain Monte Carlo settings
☒	☐	For hierarchical and complex designs, identification of the appropriate level for tests and full reporting of outcomes
☒	☐	Estimates of effect sizes (e.g. Cohen's d, Pearson's r), indicating how they were calculated

Our web collection on statistics for biologists contains articles on many of the points above.

Software and code

Policy information about availability of computer code

Data collection	RNA sequencing data was produced using standard Illumina equipment and protocols.
Data analysis	Custom software:<br>ASEReadCounter* (github.com/gimelbrantlab/asereadcounter_star); Qllelic v0.3.2 (github.com/gimelbrantlab/Qllelic).<br>Public open source software:<br>Picard (v.2.8.0; broadinstitute.github.io/picard); samtools (v.1.3.1); STAR aligner (v.2.5.4a); MBASED (v1.20.0); GeneiASE (v1.0.1); Kallisto (v.0.45.1)

For manuscripts utilizing custom algorithms or software that are central to the research but not yet described in published literature, software must be made available to editors and reviewers. We strongly encourage code deposition in a community repository (e.g. GitHub). See the Nature Research guidelines for submitting code & software for further information.

Data

Policy information about availability of data

All manuscripts must include a data availability statement. This statement should provide the following information, where applicable:

- Accession codes, unique identifiers, or web links for publicly available datasets
- A list of figures that have associated raw data
- A description of any restrictions on data availability

Data Availability statement contains this and other information: GEO record: GSE143310, https://www.ncbi.nlm.nih.gov/geo/query/acc.cgi?acc=GSE143310

Field-specific reporting

Please select the one below that is the best fit for your research. If you are not sure, read the appropriate sections before making your selection.

[x] Life sciences [ ] Behavioural & social sciences [ ] Ecological, evolutionary & environmental sciences

For a reference copy of the document with all sections, see nature.com/documents/nr-reporting-summary-flat.pdf

Life sciences study design

All studies must disclose on these points even when the disclosure is negative.

Sample size	No ab initio sample-size calculation was performed. The standard in the field is to use one replicate. The main dataset generated in the paper contains 18 technical replicates from the same RNA used to establish that the current practice is insufficient.
Data exclusions	No data was completely excluded from the analysis.<br>Replicate #1 in NEBnext experiment was considered being an outlier forming a distinct cluster in pairwise QCC calculations (discussed in the text) and it is specifically mentioned in figures' captions when it was removed.
Replication	The paper is focused on studying reproducibility in allele-specific expression measurements.<br>The main dataset analysed in the paper contains 18 technical replicates from 129xCastF1 mouse kidney total RNA.
Randomization	No randomization was used, since each of the experimental groups was determined by particular experiment: study, organism, sample, RNA, RNA-seq library and sequencing run.
Blinding	No blinding was used. Certain knowledge that replicate data come from the same biological sample is instrumental in estimation of technical noise .

Reporting for specific materials, systems and methods

We require information from authors about some types of materials, experimental systems and methods used in many studies. Here, indicate whether each material, system or method listed is relevant to your study. If you are not sure if a list item applies to your research, read the appropriate section before selecting a response.

Materials & experimental systems

n/a	Involved in the study	
[x]	[ ]	Antibodies
[ ]	[x]	Eukaryotic cell lines
[x]	[ ]	Palaeontology and archaeology
[ ]	[x]	Animals and other organisms
[x]	[ ]	Human research participants
[x]	[ ]	Clinical data
[x]	[ ]	Dual use research of concern

Methods

n/a	Involved in the study	
[x]	[ ]	ChIP-seq
[x]	[ ]	Flow cytometry
[x]	[ ]	MRI-based neuroimaging

Eukaryotic cell lines

Policy information about cell lines

Cell line source(s)	mouse v-Abl pro-B cells; clones Abl.1, Abl.2 (generated in our lab, see ref. 7)
Authentication	Cell line authentication was performed by microscopy as abelson clones have a distinct morphology. Further authentication for monoclonality was done by performing RNA-seq and droplet digital PCR on X-chromosome inactivated genes.
Mycoplasma contamination	Cell lines are regularly tested in the lab for mycoplasma contamination using MycoAlert mycoplasma detection kit (cat no. LT07-218). No indication of contamination was observed.
Commonly misidentified lines (See ICLAC register)	Not used in this study.

Animals and other organisms

Policy information about studies involving animals; ARRIVE guidelines recommended for reporting animal research

Laboratory animals	F1 breeding was performed at the DFCI mouse facility, with parent animals obtained from the Jackson Laboratories. All animal work was performed under DFCI protocol 09-065, approved by the DFCI Institutional Animal Care and Use Committee. Animals were

	housed in accordance with Guide for the Care and Use of Laboratory Animals
Wild animals	No wild animals were used.
Field-collected samples	No field-collected samples were used in this study.
Ethics oversight	The mouse work was performed under the protocol 09-065, as approved by the DFCI Institutional Animal Care and Use Committee.

Note that full information on the approval of the study protocol must also be provided in the manuscript.
